# SARS-CoV2 evokes structural brain changes resulting in declined executive function

**DOI:** 10.1371/journal.pone.0298837

**Published:** 2024-03-12

**Authors:** Daniel Deuter, Katharina Hense, Kevin Kunkel, Johanna Vollmayr, Sebastian Schachinger, Christina Wendl, Andreas Schicho, Claudia Fellner, Bernd Salzberger, Florian Hitzenbichler, Judith Zeller, Veronika Vielsmeier, Frank Dodoo-Schittko, Nils Ole Schmidt, Katharina Rosengarth

**Affiliations:** 1 Klinik und Poliklinik für Neurochirurgie, University Hospital Regensburg, Regensburg, Germany; 2 Institut für Röntgendiagnostik, University Hospital Regensburg, Regensburg, Germany; 3 Institut für Neuroradiologie, Medbo Bezirksklinikum Regensburg, Regensburg, Germany; 4 Abteilung für Krankenhaushygiene und Infektiologie, University Hospital Regensburg, Regensburg, Germany; 5 Klinik und Poliklinik für Innere Medizin II, University Hospital Regensburg, Regensburg, Germany; 6 Klinik und Poliklinik für Hals-Nasen-Ohren-Heilkunde, University Hospital Regensburg, Regensburg, Germany; 7 Institut für Sozialmedizin und Gesundheitsforschung, Otto von Guericke University Magdeburg, Magdeburg, Germany; Harvard Medical School, UNITED STATES

## Abstract

**Background:**

Several research has underlined the multi-system character of COVID-19. Though effects on the Central Nervous System are mainly discussed as disease-specific affections due to the virus’ neurotropism, no comprehensive disease model of COVID-19 exists on a neurofunctional base by now. We aimed to investigate neuroplastic grey- and white matter changes related to COVID-19 and to link these changes to neurocognitive testings leading towards a multi-dimensional disease model.

**Methods:**

Groups of acutely ill COVID-19 patients (n = 16), recovered COVID-19 patients (n = 21) and healthy controls (n = 13) were prospectively included into this study. MR-imaging included T1-weighted sequences for analysis of grey matter using voxel-based morphometry and diffusion-weighted sequences to investigate white matter tracts using probabilistic tractography. Comprehensive neurocognitive testing for verbal and non-verbal domains was performed.

**Results:**

Alterations strongly focused on grey matter of the frontal—basal ganglia—thalamus network and temporal areas, as well as fiber tracts connecting these areas. In acute COVID-19 patients, a decline of grey matter volume was found with an accompanying diminution of white matter tracts. A decline in executive function and especially verbal fluency was found in acute patients, partially persisting in recovered.

**Conclusion:**

Changes in gray matter volume and white matter tracts included mainly areas involved in networks of executive control and language. Deeper understanding of these alterations is necessary especially with respect to long-term impairments, often referred to as ‘Post-COVID’.

## Introduction

Coronavirus disease 2019 (COVID-19) is caused by the severe acute respiratory syndrome coronavirus 2 (SARS-CoV-2), which was first identified in Wuhan, China [1], rapidly spreading around the world causing a global pandemic in the following. Though SARS-CoV-2 is mainly attributed to infect the respiratory system causing severe pneumonia, a multitude of studies is rather underlining a multi-system character of COVID-19 including involvement of the digestive system, the cardiovascular system and the central nervous system (CNS) [2, 3].

Neurological symptoms in COVID-19 patients include moderate to severe impairments like headache, reduced consciousness, stroke, seizure, encephalitis, taste- and smell dysfunction, fatigue and sleep disturbances [4, 5]. Regarding structural disease-related CNS-alterations, brain abnormalities were reported in COVID-19 patients including an increased signal in the area of the olfactory bulb and the olfactory tract, but also white matter hyperintensities [6]. Using Diffusion Tensor Imaging (DTI) and T1 weighted images, changes in grey matter volume were found in specific areas as well as differences in fractional anisotropy (FA) and mean diffusivity (MD) in a variety of subcortical fiber tracts in previous studies [7–10].

Possible effects of these structural changes could be impairments in various neurocognitive functions as reported in previous studies. Significant deficits were found in COVID-19 patients in the areas of attention [11, 12], memory [11, 13], executive functions [14, 15], visuospatial processing [14, 15] and language [11, 14]. Those neurocognitive symptoms often outlast the phase of acute illness by a multiple and can lead to long-term impairments, often referred to as ‘Post-COVID’, continuing to limit the lives of affected patients even months after recovery [13, 16, 17].

Pathophysiologically, the spike-like protein (S-protein) of SARS-CoV-2 binds to the Angiotensin-converting enzyme 2 (ACE2) receptor of human cells and thus enters the cell. Since ACE2 is not only present in the airway epithelia and lung parenchyma, but also in other tissues such as the vascular endothelium or glial cells and neurons of the brain, this could explain not only typical respiratory tract-associated symptoms, but also neurological and neurocognitive symptoms. Nevertheless, different mechanisms like migration through the cribriform plate, inflammatory-induced spread through the blood brain barrier and trans-synaptic migration are also discussed as possible correlates enabling the virus’ neurotropism [4, 18, 19].

Even if the synopsis of the existing literature clearly points towards a disease-specific CNS-affection, no comprehensive disease model of COVID-19 exists on a functional base by now. Especially the question whether certain cerebral networks are more affected by COVID-19 related sequelae than others and how other areas of the brain adopt to these changes, hasn’t been completely understood yet.

The aim of our study was to investigate neuroplastic grey- and white matter changes related to COVID-19 and to link these structural changes to comprehensive neuropsychological and neurocognitive testings. For the combination of these morphological, functional and temporal parameters, eventually leading towards a comprehensive multi-dimensional whole-brain model, patients with acute COVID-19 infection and patients recovered from COVID-19 were compared to healthy control subjects.

## Methods

This study was approved by the local ethics committee of the University of Regensburg (protocol code 20-1831-101) and was performed in accordance with the Declaration of Helsinki. Written informed consent was obtained from each subject.

### Patients’ cohort

Initially, a total of 52 subjects was prospectively recruited for this study between May 2020 and April 2021. Two acutely ill patients were excluded from further analyses due to pre-existing cerebral pathologies (one subject with stroke in patient’s history, one subject with pronounced Virchow-Robin spaces). The group of acutely ill subjects was recruited from patients admitted to the University Hospital Regensburg requiring medical attendance due to COVID-19. Recovered patients had positive PCR testing at the University Hospital Regensburg at least 6 months ago and were actively called for recruitment. Healthy volunteers were prospectively included as controls into this study. A one-way ANOVA was used to test for age differences between the groups.

### MRI acquisition

Data collection took place between May 2020 and April 2021 at both the University Hospital Regensburg and the Neuroradiological Institute at the District Hospital of Regensburg.

Acutely ill patients admitted to the University Hospital Regensburg were examined at this study site using a 3T Siemens Skyra scanner (Siemens, Erlangen, Germany); recovered COVID-19 patients and healthy control subjects were examined at the Neuroradiological Institute at the District Hospital of Regensburg using a 1.5T Siemens Aera scanner. The reason for the use of two scanners at two study sites was to avoid clinically unnecessary transports of infectious patients between study sites and to avoid potentially health risks for control subjects in accordance with the high pandemic restrictions during this time.

A three-dimensional (3D) T1-weighted Magnetization Prepared Rapid Acquisition with Gradient Echo (MP-RAGE) sequence was acquired for the evaluation of voxel-based morphometry (VBM). For evaluation of white matter alterations, Diffusion Tensor Imaging (DTI) data were acquired with a b-value of 0/ 1000 in 30 gradient directions. To exclude cerebral diseases such as stroke, which would bias the results, a 3D FLuid Attenuated Inversion Recovery (FLAIR) and a 3D Susceptibility Weighted Image (SWI) sequence were obtained and reviewed by a neuroradiologist with many years of clinical routine. Detailed MRI parameters of each sequence acquired are shown in Table 1.

**Table 1:** MRI parameters of the acquired sequences used for the study.

	3T SIEMENS Skyra Scanner				1.5T SIEMENS Aera Scanner			
	MPR	DTI	FLair	SWI	MPR	DTI	FLair	SWI
TR	1910ms	5200ms	5000ms	27ms	2260ms	6700ms	5000ms	49ms
TE	3.67ms	95ms	386ms	20ms	5.07ms	82ms	325ms	40ms
FoV	250x250	230x230	240x240	185x220	250x250	230x230	227x260	185x220
	mm^2^	mm^2^	mm^2^	mm^2^	mm^2^	mm^2^	mm^2^	mm^2^
Flip Angle	9°	90°	120°	15°	8°	90°	120°	15°
Voxel Size (reconstructed)	0.98x0.98x1.00mm	0.90x0.90x3.00mm	0.47x0.47x0.9mm	0.86x0.86x1.6mm	0.98x0.98x1.00mm	0.90x0.90x3.00mm	0.51x0.51x1.00mm	0.69x0.69x1.60mm
Voxel Size (measured)	0.98x0.98x1.00mm	1.80x1.80x3.00mm	0.94x0.94x1.12mm	0.90x0.86x1.60mm	0.98x0.98x1.00mm	1.80x1.80x3.00mm	1.04x1.04x1.26mm	0.81x0.69x2.01mm
Slice Thickness	1.00mm	3.00mm	0.9mm	1.6mm	1.00mm	3.00mm	1.00mm	1.60mm

Abbreviations: DTI = Diffusion Tensor Imaging, FLair = FLuid Attenuated Inversion Recovery, FoV = Field-of-view, MPR = MP-RAGE, SWI = Susceptibility Weighted Images, TE = echo time, TR = repetition time.

To minimize relevant scanner artifacts, an additional validation cohort consisting of another 13 healthy volunteers was examined at both MRI scanners used in this study after the end of the pandemic restrictions. Data from this validation cohort was used to adjust the data from the study cohorts between the two scanners. For each subject from this validation cohort, tract- and ROI-specific quotients between the two validational scans were calculated for subsequent analyses as specified below (see “Voxel-based morphometry” and “Probabilistic tractography”). Mean quotients were determined for each tract and ROI analyzed within the main study cohort separately and used to individually correct the data from the study cohort for scanner-induced artifacts at a regional level.

### Segmentation

In order to evaluate differences in grey and white matter volume, the T1-weighted images of all subjects were segmented into grey matter, white matter and cerebrospinal fluid (CSF) using the Computational Anatomy Toolbox (CAT12; Version 12.8; dbm.neuro.uni-jena.de/cat/; last access: 05.11.2021) implemented in Statistical Parametric Mapping 12 (SPM12; Version 7771; www.fil.ion.ucl.ac.uk/spm/software/spm12/; last access: 05.11.2021). For the analysis of differences in total intracranial volume (TIV) and normalized grey matter-, white matter- and CSF volume between the three study groups, a repeated-measures ANOVA was used.

### Voxel-based morphometry

In order to evaluate differences in grey matter volume in detail, results from the segmentation with the CAT12 toolbox were compared between the different groups in a whole brain analysis in SPM12 using a full factorial design. To minimize the influence of potential confounding factors, TIV, age, and severity of COVID-19 disease were included as covariates.

To further specify the extent of the calculated differences between the three study groups, the volumes of individual Regions Of Interest (ROIs) within the significant cluster areas in the group analyses were compared separately. For this purpose, the gray matter volume was segmented into the 170 brain areas of the AAL3-atlas (Automated anatomical labeling atlas version 3v1) [20] as implemented in the CAT12 toolbox with the subdivided regions of thalamus, cerebellum and vermis summed up. Differences in grey matter volume of the individual ROIs were calculated in a MANCOVA using the patients’ age as a covariate.

To minimize effects of general brain size (e.g. due to sex differences between groups) and to enable comparisons of the results of the whole brain analysis and those of the individual ROIs, the individual absolute volumes of grey and white matter were normalized. For the whole brain analysis, normalization of the data into the standard MNI space (Montreal Neurological Institute) was performed as implemented in SPM12. When analyzing the gray matter volumes using the CAT12 toolbox, the TIV, calculated in the individual space, was compared for each patient with the average TIV in the standard space. Subsequently, the individual TIV was multiplied with the ratio of the deviation. Since the volumes of ROIs were also calculated in the individual space, this ratio was also used for the normalization of all other ROIs, as the volumes would otherwise be biased by different sizes and volumes of the entire brain, such as those between men and women [21]. As sex was already taken into account in the processing pipeline, this was no longer included as a covariate in later analyses.

To additionally minimize scanner-induced influences on the results, data was adjusted based on the validation cohort examined at both scanners. Each subject from this validation cohort was examined on both scanners and all analyses were performed as described above for the main study cohort. After processing of MRI data from both scanners, quotients of the mean volumes from the two scanners were calculated for each ROI separately and used to adjust the raw VBM data from the 3T scanner.

As gray matter volume is also dependent on factors like cortical thickness and total surface area, further ROI analyses on cortical thickness were performed. For this purpose, cortical thickness values were calculated analogously to the gray matter volume using the CAT12 toolbox. The individual segments of the Destrieux Atlas [22] were used as ROIs. Data was also adjusted using the quotients from the validation cohort.

### Probabilistic tractography

Probabilistic tractography was performed using FSL 6.0.3 (https://fsl.fmrib.ox.ac.uk/fsl/fslwiki/; last access: 05.11.2021) [23]. After conversion of DICOM files into the NIFTI format using the dcm2-nii tool (people.cas.sc.edu/rorden/mricron/; last access: 05.11.2021), data were corrected for susceptibility-induced distortions based on the combination of image acquisitions with opposing polarities of the phase-encode blips (anterior–posterior direction, “A2P”, and posterior–anterior direction, “P2A”) as well as for proband movement errors and eddy currents using TOPUP and EDDY [24, 25]. Skull stripping was performed using BET [26]. Probability distributions of diffusion parameters were calculated with BedpostX (“Bayesian Estimation of Diffusion Parameters Obtained using Sampling Techniques” with X representing the possibility of modelling crossing fibers). Using this approach, probability models of various diffusion orientations rather than just calculating one mean diffusion vector for each voxel were computed running Markov Chain Monte Carlo sampling [27, 28]. Subsequently, streamlines connecting predefined seed- and target regions were iteratively calculated based on these voxel-wise distributions [27, 28]. For objective and automatized fiber tracking regarding a variety of important fiber tracts, XTRACT was used [29]. XTRACT utilizes seed- and target masks based on atlases defined in the MNI152 standard space derived from 1021 subjects from the Human Connectome Project [29]. Therefore, individual patient data are warped into the standard space, atlas files are co-registered with the individual anatomy and transformed back into the native patient space. Afterwards, probabilistic tractography is performed in the native patient space individually for each patient for 42 tracts including the Anterior Commissure (AC), Arcuate Fasciculus (AF), Acoustic Radiation (AR), Anterior Thalamic Radiation (ATR), dorsal/ peri-genual and temporal Cingulum subsection (CBD, CBP, CBT), Corticospinal Tract (CST), Frontal Aslant (FAs), Forceps Major and Minor (FMA, FMI), Fornix (FX), Inferior Longitudinal Fasciculus (ILF), Inferior Fronto-Occipital Fasciculus (IFO), Middle Cerebellar Peduncle (MCP), Middle Longitudinal Fasciculus (MdLF), Optic Radiation (OR), Superior Thalamic Radiation (STR), Superior Longitudinal Fasciculus 1/ 2/ 3 (SLF1, SLF2, SLF3), Uncinate Fasciculus (UF) and Vertical Occipital Fasciculus (VOF).

Statistics including the individual tract’s volume, mean/ median tract probability, tract length, FA and MD including standard deviations were extracted for each tract. Tract volumes and tract lengths were normalized to the individual brain volume derived from T1 brain masks calculated by BET. Tracts where fiber tracking using XTRACT was not possible (tract volume = 0) were excluded from further analyses. Mean values of the above mentioned parameters were calculated for each tract over all subjects of the individual group.

To minimize scanner-based artifacts, data was adjusted based on the validation cohort examined at both scanners. For the subjects of the validation cohort, all analyses were performed as described above for the main study cohort for both scanners. Quotients of the parameters of interest (normalized tract volume, normalized tract length, FA and MD) were calculated for each tract separately between the two scanners and used to adjust the raw tractographic data from the 3T scanner.

Additionally, voxelwise statistical analysis of the FA and MD data was performed using Tract-Based Spatial Statistics (TBSS) [23, 30]. Using this approach, brain-extracted FA images were transformed into a common space using non-linear registration [31, 32] to calculate a mean FA skeleton of all tracts on a whole-brain base. Afterwards, voxelwise cross-subject statistics were performed for the FA and MD data of each individual subject projected onto this mean FA skeleton. TFCE (Threshold-Free Cluster Enhancement) was used for voxelwise statistics. Analyses were performed on raw data not adjusted based on the results from the validation cohort examined at both scanners.

### Neuropsychological assessment

Verbal memory was assessed with the Logical Memory I / II test included in the Wechsler Adult Intelligence Scale (WAIS) [33]. We tested for verbal working memory and attention with the Number Span Forward/ Backward test also implemented in the WAIS. The Rey Complex Figure test [34] was used to test for visuo-constructive functions and visual memory, while visual working memory was examined using the Corsi Block Span task [35]. Visuo-motor speed and executive functions were examined using the Trail Making Test [36]. In addition, the Regensburg Word Fluency Test (RWAT) [37] was used to examine lexical and semantic word fluency. To test for attention and visuo-motor speed, the Digit Symbol Coding Test [33] was used. Sociodemographic data was collected for adequate analysis of the specific tests according to the instructions of the individual tests.

Because of the lack of comparability of tests’ raw scores for example due to the strong dependence on the age of the subjects, scores were transformed into z-scores based on the test instructions and further processed into percentile ranks on the basis of the respective norm sample taking the age of the subject into account. In the case of the Regensburg Word Fluency Test, the percentile ranks were determined directly from the raw scores. As percentile ranks already depend on the patient’s age, no further adjustment for age between the study groups was performed.

### Statistics

The numerical data was evaluated using SPSS Version 25 and 26 (IBM, Armonk, USA). For repeated measures ANOVA, Mauchly’s test of sphericity was performed to assess the equal variances of the differences between the within-subject factors. In case of violations of sphericity, we used the Greenhouse–Geisser adjustment.

Regarding VBM, for all brain areas involved in statistically significant clusters in the whole brain analysis, post-hoc t-tests were performed using Benjamini-Hochberg correction [38] for multiple testing (P_adj._ < .05) after adjustment of results based on the quotients calculated from the results of the validation cohort. To test for statistically significant differences between the groups regarding the results from probabilistic tractography after adjustment between scanners, one-way ANOVAs were computed with post-hoc t-tests corrected with Bonferroni correction for multiple testing (*P*_*adj*._ < .05).

To investigate differences between the three study groups regarding their scores on the various neuropsychological tests, results were examined using the Kruskal-Wallis test for unpaired samples.

## Results

### Patients’ characteristics

**Fig 1:**
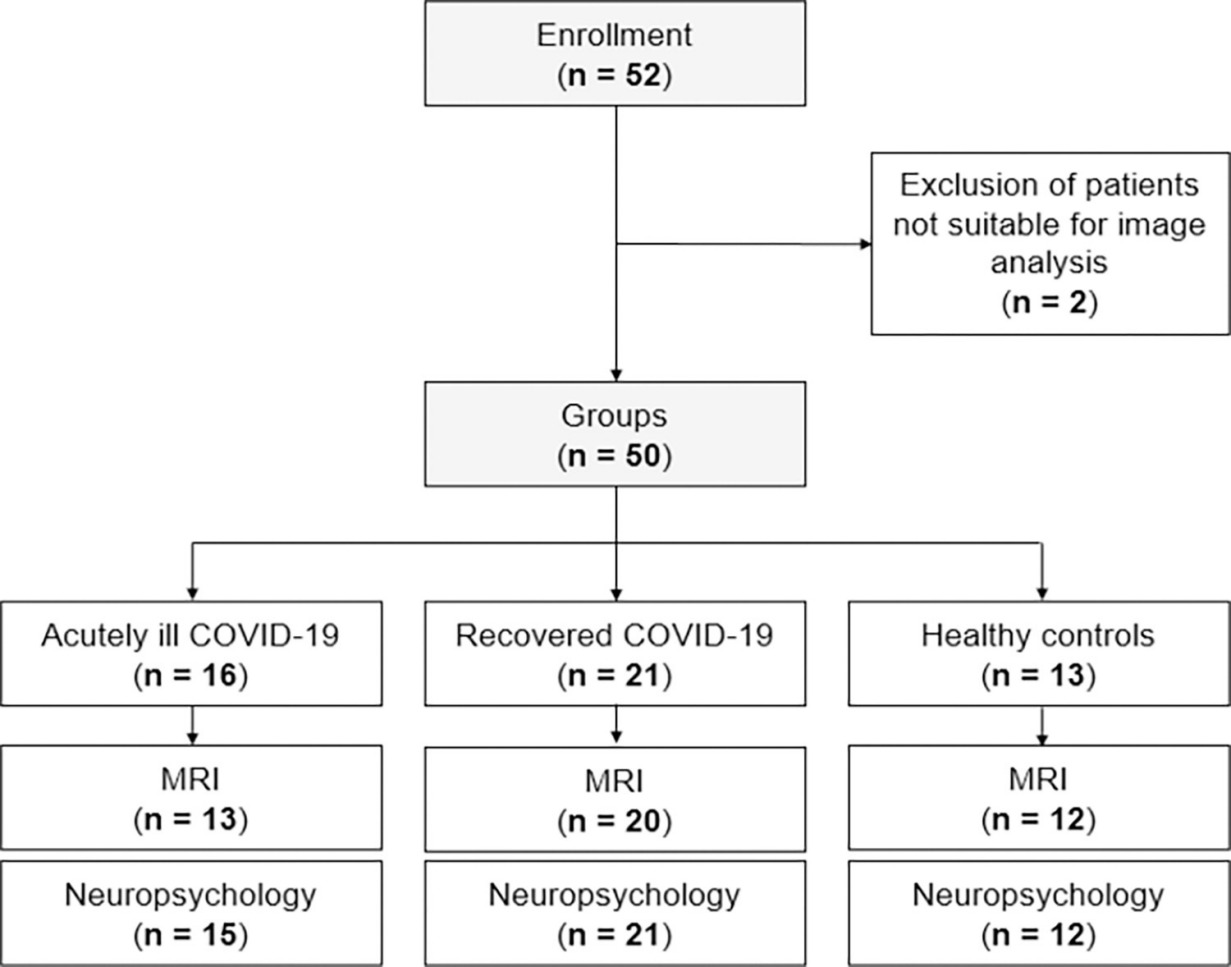
Participants’ flow chart. Two acutely ill patients were excluded from further analyses due to pre-existing cerebral pathologies (one subject with stroke in patient’s history, one subject with pronounced Virchow-Robin spaces). Abbreviations: MRI = magnetic resonance imaging.

The cohort consisted of 16 acutely ill COVID-19 patients, 21 recovered COVID-19 patients and 13 healthy controls (Fig 1, Table 2). A one-way ANOVA used to test for age differences between the groups showed that the groups differed significantly with respect to age (F(2,48) = 6.556; *P* = .003). Post hoc t-tests (FDR-corrected) revealed that the group of acutely ill patients differed from that of recovered patients (*P*_*adj*._ = .001) and healthy control subjects (*P*_*adj*._ = .003). Between recovered patients and healthy controls, no age difference was found (*P*_*adj*._ = .994).

**Table 2:** Composition and characteristics of the patients’ cohort including demographic and clinical information on included subjects.

	Acute COVID-19 patients	Recovered COVID-19 patients	Healthy controls
**Sample size n**	16	21	13
**Mean age**	53.75	39.65	39.62
**Sex**	13 (81.2%) male	10 (47.6%) male	6 (46.2%) male
	3 (18.8%) female	11 (52.4%) female	7 (52.8%) female
**Disease severity**	6 (37.5%) mild	20 (95.2%) mild	13 (100%) no disease
	5 (31.3%) moderate		
	3 (18.8%) severe	1 (4.8%) moderate	
	2 (12.5%) critical		
**MRI**	13 (81.3%)	20 (95.2%)	12 (92.3%)
**Neuropsychological assessment**	15 (93.8%)	21 (100%)	12 (92.3%)
**ICU**	3 (18.8%) patients	0 patients	/
**Anosmia**	2 (12.5%) anosmia	13 (61.9%) anosmia	/
	14 (87.5%) no anosmia	8 (38.1%) no anosmia	
**Dysgeusia**	2 (12.5%) dysgeusia	11 (52.4%) dysgeusia	/
	14 (87.5%) no dysgeusia	10 (47.6%) no dysgeusia	

Abbreviations: MRI = magnetic resonance imaging, ICU = Intensive Care Unit.

### Segmentation

A repeated-measures ANOVA used for the comparison of TIV, normalized grey matter-, white matter- and CSF volume between the three study groups adjusting for the variable Age as a covariate, revealed a significant main effect of the inner-subject factor Tissue (TIV/ grey matter volume/ white matter volume/ CSF volume; F(1.667,88.349) = 98.018; *P* < .001), a significant interaction effect Tissue x Age (F(1.667,88.349) = 9.694; *P* < .001) and a trend in the interaction Tissue x Group (F(3.334,88.349) = 2.225; *P* = .084). No significant main effects were observed for the between-subject factors Age (F(1,53) = .246; *P* = .622) and Group (F(2,53) = .578; *P* = .565).

**Fig 2:**
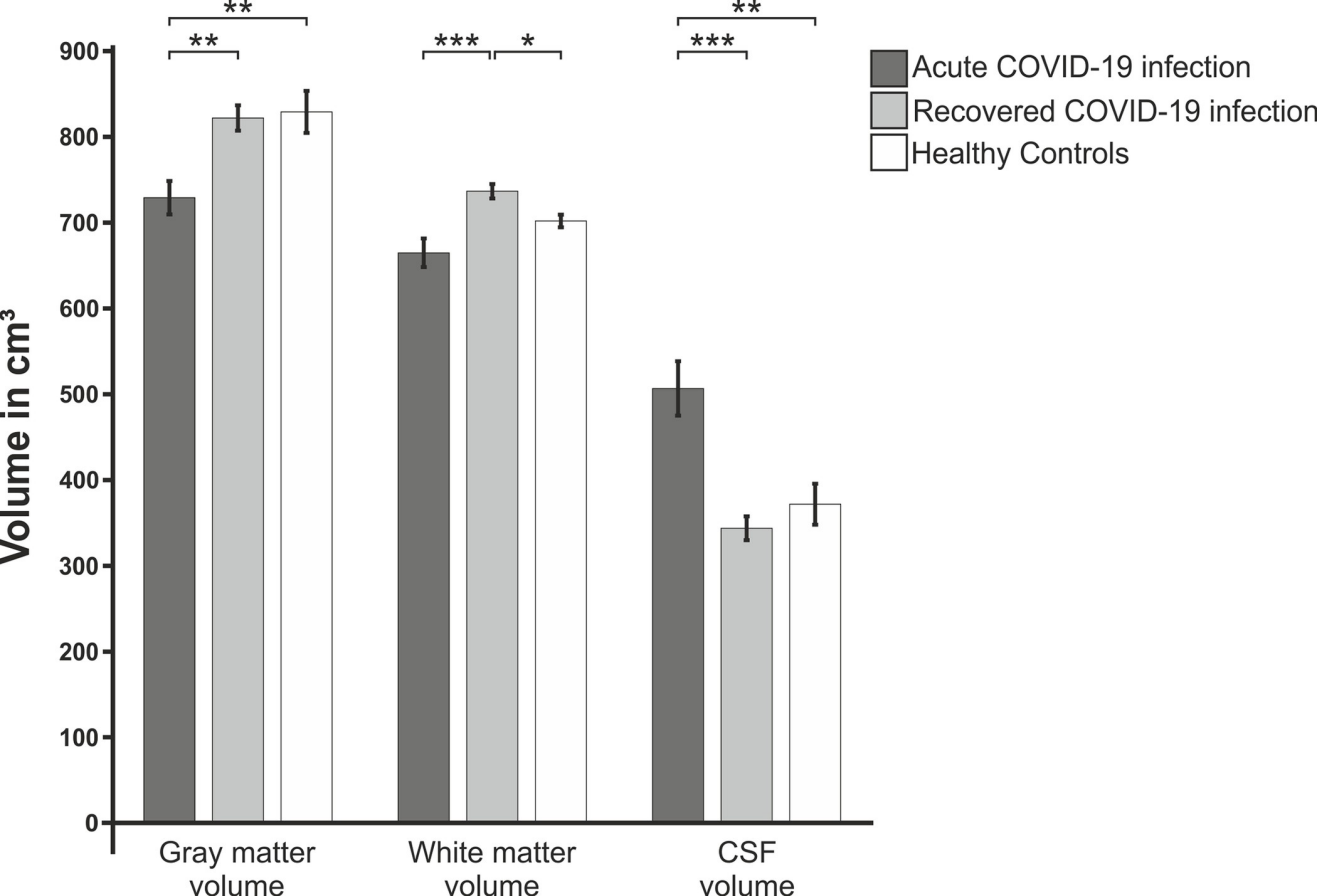
General differences in tissue volume between the three study groups. The volumes of grey matter, white matter and CSF were normalized using the total intracranial volume before analysis in order to avoid systematic differences between groups due to different brain size. Dark-grey columns represent acute COVID-19 patients, grey columns represent recovered COVID-19 patients, white columns represent healthy controls. Statistically significant results were marked with * for P < .05, ** for P < .01 and *** for P < .001.

Post-hoc t-tests showed a significant difference in grey matter volume between acutely ill patients and recovered patients (*P*_*adj*._ = .002) as well as acutely ill patients and healthy controls (*P*_*adj*._ = .007, Fig 2). White matter volume significantly differed between acutely ill patients and recovered patients (*P*_*adj*._ < .001) as well as recovered patients and healthy controls (*P*_*adj*._ = .013). Differences in CSF volume were significant between acutely ill patients and recovered patients (*P*_*adj*._ < .001) as well as acutely ill patients and healthy controls (*P*_*adj*._ = .006).

Pearson’s correlations to investigate the influence of age on the volumes of the specific compartments showed significant bivariate correlations between age and grey matter volume (r = -.827; *P* < .001) as well as age and CSF volume (r = .747; *P* < .001). A trend was found for age and white matter volume (r = -.264; *P* = .080).

### Voxel-based morphometry

In the following, results on differences in grey matter volumes between the three study groups are presented a) from the whole-brain analysis using SPM12 including TIV, age, and severity of COVID-19 disease as covariates and b) from the more detailed analysis of individual ROIs inside the significant cluster areas in a repeated measures ANOVA including the patients’ age as a covariate. Results from the validation cohort used to adjust the data of the individual ROIs in order to minimize artifacts between the different MRI scanners are shown in S1 Table in S1 File.

**Fig 3:**
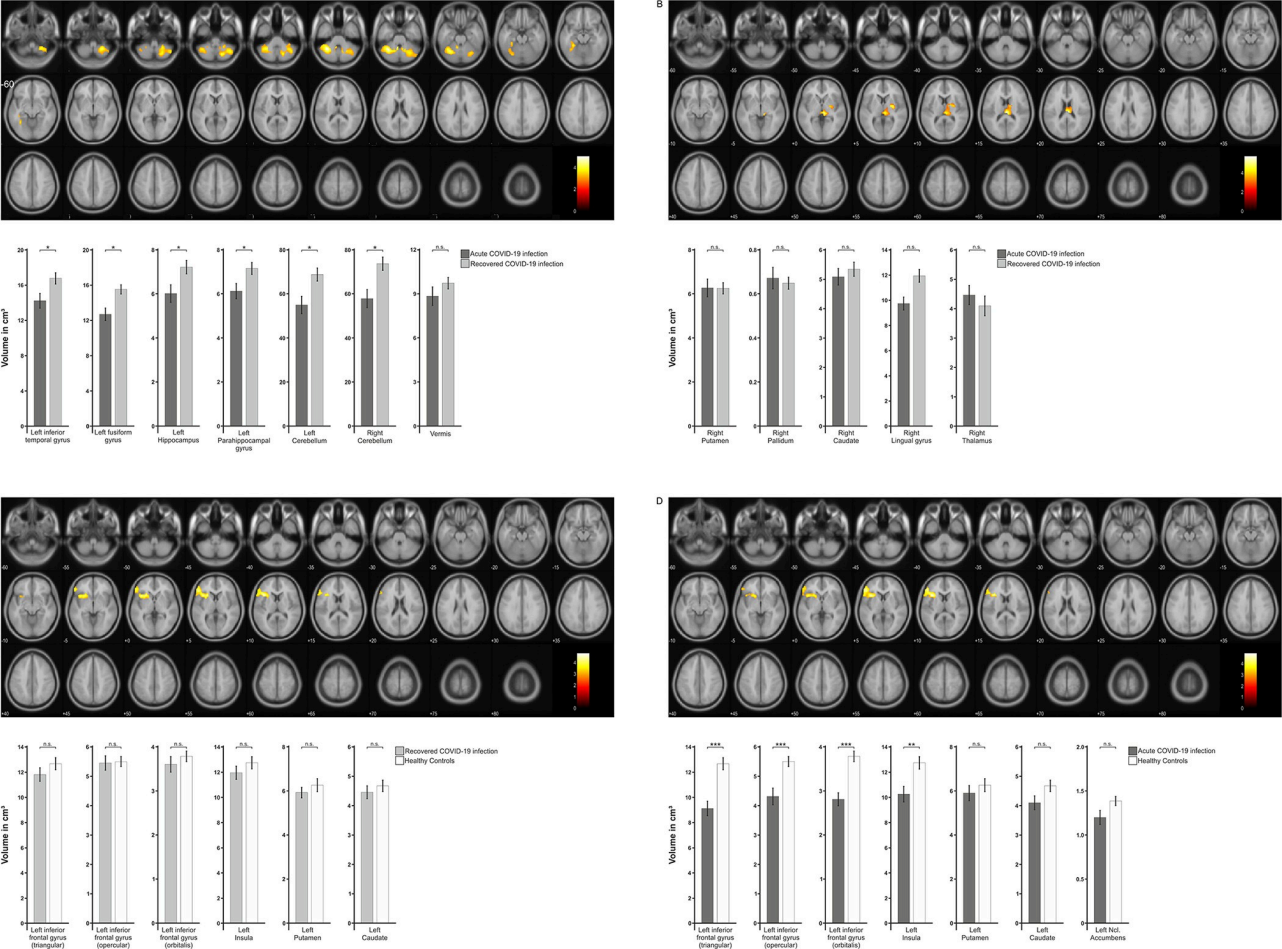
Results of whole-brain grey matter analyses and corresponding ROI analyses of labels included in significant clusters. Clusters of higher grey matter volumes (t = 3) in recovered patients compared with acutely ill patients (**A**), in acutely ill patients compared with recovered patients (**B**), in healthy controls compared with recovered patients (**C**), and in healthy controls compared with acutely ill patients (**D**) are presented. Areas involved in the significant clusters were subsequently examined separately in post-hoc t-tests. The subareas of the right thalamus, left cerebellum, right cerebellum, and vermis originally subdivided in the AAL3 atlas were combined for post-hoc analyses. Results are presented in diagrams below (dark grey: acute COVID-19 patients, grey: recovered COVID-19 patients, white: healthy controls). Statistically significant results were marked with * for P < .05, ** for P < .01 and *** for P < .001. Error bars represent ±1 SEM.

When comparing acutely ill and recovered patients on a whole-brain approach, recovered patients showed clusters of significantly higher grey matter volumes in several parts of both hemispheres of the cerebellum as well as in the fusiform gyrus, inferior temporal gyrus, parahippocampal gyrus, and the hippocampus (Fig 3A). Subsequent post-hoc unpaired t-tests (FDR-corrected) of the individual ROI volumes revealed significant differences between the two groups in the left inferior temporal gyrus (t(31) = -2.459; *P*_*adj*._ = .028), left fusiform gyrus (t(31) = -3.296; *P*_*adj*._ = .011), left hippocampus (t(31) = -2.424; *P*_*adj*._ = .028), left parahippocampal gyrus (t(31) = -2.368; *P*_*adj*._ = .028), left cerebellum (t(31) = -2.866; *P*_*adj*._ = .016), and right cerebellum (t(31) = -3.185; *P*_*adj*._ = .011), but not in the vermis (t(31) = -1.265; *P*_*adj*._ = .215). In addition, whole-brain analysis showed that areas in the thalamus, lingual gyrus, and basal ganglia had higher volume in acutely ill patients (Fig 3B). Post-hoc t-tests did not show any significant differences in the right thalamus (t(31) = .752; *P*_*adj*._ = .874), the right pallidum (t(31) = .450; *P*_*adj*._ = .874), right putamen (t(31) = -.688; *P*_*adj*._ = .874) and right caudate (t(31) = -.038; *P*_*adj*._ = .970).

Second, healthy controls were compared with recovered COVID-19 patients. The whole-brain analysis showed significant differences in grey matter volume in the inferior frontal gyrus, insula and basal ganglia (Fig 3C). Post-hoc t-tests of the individual ROI volumes, however, showed no significant differences for the left inferior frontal gyrus pars triangularis (t(30) = -1.089; *P*_*adj*._ = .601), left inferior frontal gyrus pars opercularis (t(30) = -.146; *P*_*adj*._ = .885), left inferior frontal gyrus pars orbitalis (t(30) = -.739; *P*_*adj*._ = .601), left insula (t(30) = -1.024; *P*_*adj*._ = .601), left putamen (t(30) = -.874; *P*_*adj*._ = .601), and left caudate (t(30) = -.681; *P*_*adj*._ = .601).

When comparing healthy controls to acutely ill patients, the whole-brain analysis showed a similar pattern as found in the comparison with recovered COVID-19 patients. Again, significant differences were found in the inferior frontal gyrus, insula, basal ganglia, and additionally in the nucleus accumbens (Fig 3D). Post-hoc t-tests of the individual ROIs showed significant differences in the left inferior frontal gyrus pars triangularis (t(23) = -4.695; *P*_*adj*._ < .001), left inferior frontal gyrus pars opercularis (t(23) = -3.516; *P*_*adj*._ = .004), left inferior frontal gyrus pars orbitalis (t(23) = -5.085; *P*_*adj*._ < .001), left insula (t(23) = -3.157; *P*_*adj*._ = .008), while left caudate (t(23) = -1.867; *P*_*adj*._ = .087), and left nucleus accumbens (t(23) = -1.929; *P*_*adj*._ = .087) showed a trend and the left putamen (t(23) = -.793; *P*_*adj*._ = .436) showed no significant difference.

Detailed results of whole-brain and ROI analyses are presented in S2 and S3 Tables in S1 File, detailed results of the ROI analyses after adjustment for age and scanner effect can be found in S4 Table in S1 File. Results from the ROI analyses on cortical thickness showed wide-spread reductions in cortical thickness especially in acutely ill patients (S5 Table in S1 File).

### Probabilistic tractography

**Fig 4:**
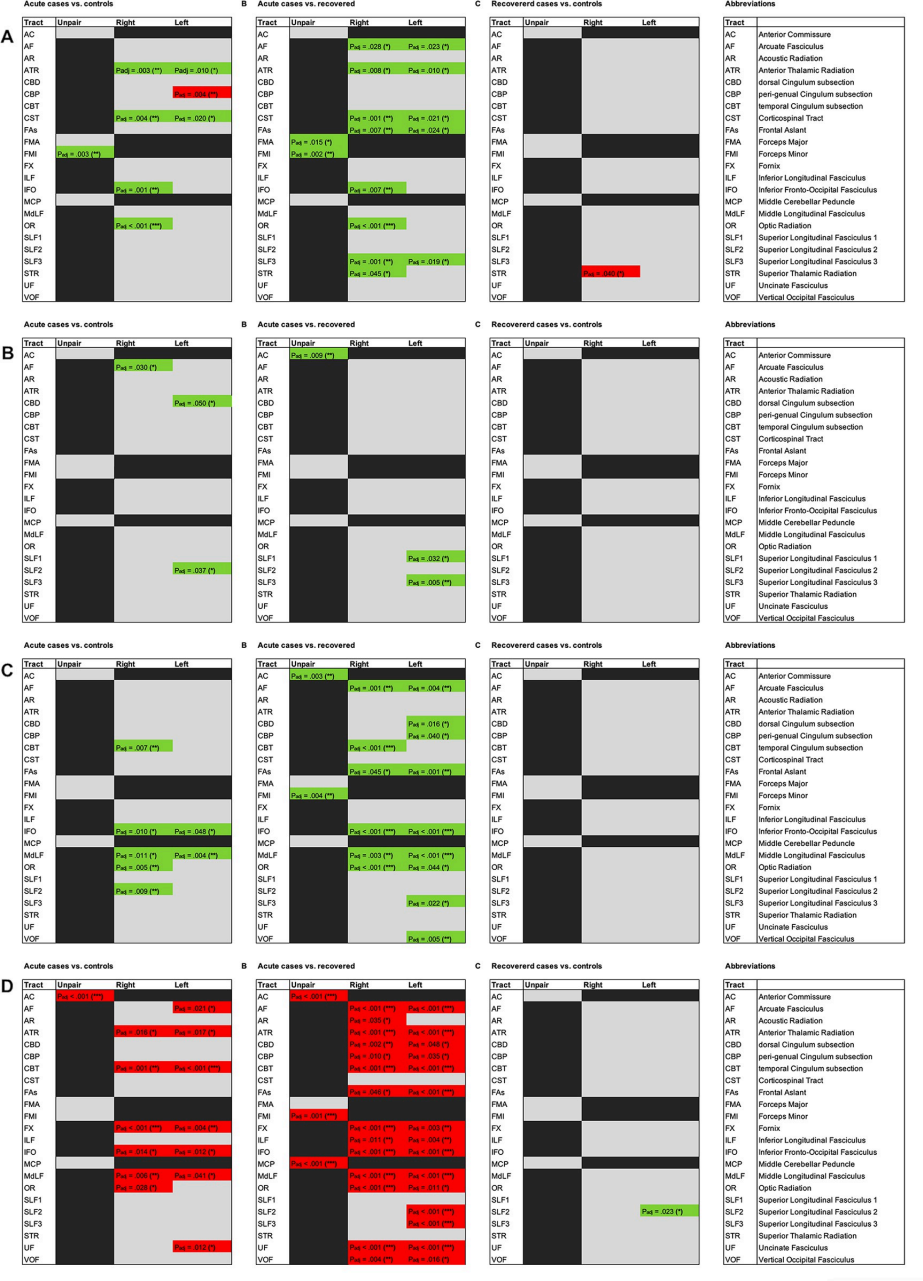
Matrices of significant differences regarding white matter changes between groups. (**A**) normalized tract volume; (**B**) normalized tract lengths; (**C**) fractional anisotropy (FA); (**D**) mean diffusivity (MD). In each row, significant differences between (**I**) acute cases vs. controls; (**II**) acute cases vs. recovered; (**III**) controls vs. recovered cases, are presented. Green fields indicate significant differences of mean values with positive direction between the groups, red fields indicate significant differences of mean values with negative direction between the groups. P values corrected for multiple testing are presented. Statistically significant results were marked with * for P < .05, ** for P < .01 and *** for P < .001. Abbreviations of the presented tracts are explained in the right column.

Probabilistic tractography was used to evaluate differences in white matter and subcortical fiber tracts based on DTI sequences. Summarizing matrices of all significant findings regarding the specific tracts are presented in Fig 4. Results of the validation cohort used for normalization between the scanners are shown in S1 to S4 Figs in S1 File.

#### Normalized tract volume

Comparing acute cases vs. controls, significant differences were found for ATR bilateral (*P*_*adj*._ = .010 left, *P*_*adj*_ = .003 right), CST bilateral (*P*_*adj*._ = .020 left, *P*_*adj*_ = .004 right), CBP left (*P*_*adj*._ = .004), IFO right (*P*_*adj*._ = .001), OR right (*P*_*adj*._ < .001) and FMI (*P*_*adj*._ = .003) with lower volumes in acute cases except from CBP left (Fig 4A and S6 Table S1 File). Comparing acute cases vs. recovered cases, significant differences were found for AF bilateral (*P*_*adj*._ = .023 left, *P*_*adj*_ = .028 right), ATR bilateral (*P*_*adj*._ = .010 left, *P*_*adj*_ = .008 right), CST bilateral (*P*_*adj*._ = .021 left, *P*_*adj*_ = .001 right), FAs bilateral (*P*_*adj*._ = .024 left, *P*_*adj*_ = .007 right), SLF3 bilateral (*P*_*adj*._ = .019 left, *P*_*adj*_ = .001 right), IFO right (*P*_*adj*._ = .007), OR right (*P*_*adj*._ < .001), STR right (*P*_*adj*._ = .045), FMA (*P*_*adj*._ = .015) and FMI (*P*_*adj*._ = .002). with lower volumes in acute cases. Comparing recovered cases with controls, except from STR right (*P*_*adj*._ = .040), no significant differences were found (Fig 4A and S6 Table S1 File).

#### Normalized tract length

Comparing acute cases vs. controls, significant differences were found for SLF2 left (*P*_*adj*._ = .037), CBD left (*P*_*adj*._ = .050) and AF right (*P*_*adj*._ = .030) with shorter tract lengths in acute cases (Fig 4B and S6 Table in S1 File). Comparing acute cases vs. recovered cases, significant differences were found for SLF1 left (*P*_*adj*._ = .032), SLF3 left (*P*_*adj*._ = .005) and AC (*P*_*adj*._ = .009) with shorter tract lengths in acute cases. Comparing recovered cases with controls, no significant differences were found (Fig 4B and S6 Table in S1 File).

#### Mean FA

The Fractional Anisotropy represents the directionality of water diffusion [39], ranging from values between 0 and 1 with full directionality if FA = 1. Comparing acute cases vs. controls, significant differences could be found for a variety of tracts (IFO bilateral, MdLF bilateral, CBT right, OR right, SLF2 right; Fig 4C and S7 Table in S1 File). Lower FA values could be consistently found in acute cases indicating less directionality of diffusion, probably representing less white matter integrity [40]. Comparing acute cases vs. recovered cases, significant differences were found for a variety of tracts (AF bilateral, FAs bilateral, IFO bilateral, MdLF bilateral, OR bilateral, CBD left, CBP left, SLF3 left, VOF left, CBT right, AC, FMI; Fig 4C and S7 Table in S1 File). FA values were consistently lower in acute cases. Comparing recovered cases with controls, no significant differences could be found (Fig 4C and S7 Table in S1 File).

#### Mean MD

The Mean Diffusivity represents the magnitude of water diffusion with 0 depicting no diffusion at all [39, 40]. Comparing acute cases vs. controls, significant differences were found for a variety of tracts (ATR bilateral, CBT bilateral, FX bilateral, IFO bilateral, MdLF bilateral, AF left, UF left, OR right, AC; Fig 4D and S7 Table in S1 File). Higher MD values could be consistently found in acute cases indicating higher diffusivity. Comparing acute cases vs. recovered cases, significant differences could be found for a variety of tracts (AF bilateral, ATR bilateral, CBD bilateral, CBP bilateral, CBT bilateral, FAs bilateral, FX bilateral, ILF bilateral, IFO bilateral, MdLF bilateral, OR bilateral, UF bilateral, VOF bilateral, SLF2 left, SLF3 left, AR right, AC, FMI, MCP; Fig 4D and S7 Table in S1 File). MD values were consistently higher in acute cases. Comparing recovered cases with controls, except from SLF2 left (*P*_*adj*._ = .023), no significant differences could be found (Fig 4D and S7 Table in S1 File).

Results from TBSS are shown in S5 to S7 Figs in S1 File. TBSS analyses replicated the findings of widely reduced FA and increased MD values in acute patients compared to controls as well as recovered patients in a variety of fiber tracts. Comparing controls to recovered patients, no significant results were found using TBSS.

### Neuropsychological assessment

**Fig 5:**
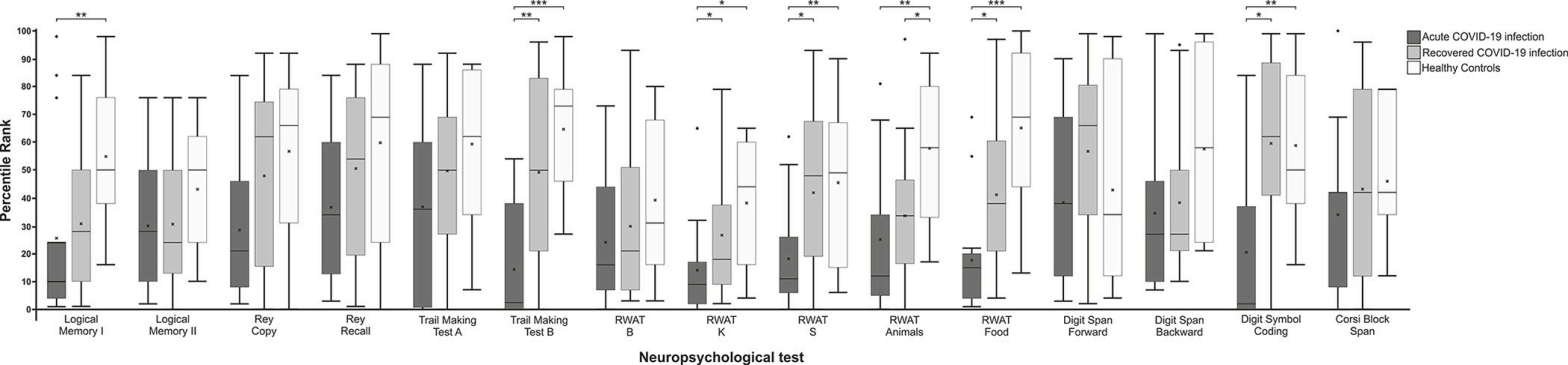
Results of the individual neuropsychological tests for each of the three study groups displayed as percentile ranks. Dark-grey columns represent acute COVID-19 patients, grey columns represent recovered COVID-19 patients, white columns represent healthy controls. Statistically significant results were marked with * for P < .05, ** for P < .01 and *** for P < .001. Error bars represent ±1 SEM. Abbreviations: RWAT = Regensburg Word Association Test.

The Kruskal-Wallis test for unpaired samples revealed significant differences in the following tests (Fig 5): The Logical Memory I test (χ^2^ (2) = 8.190; *P* = .017), the Trail Making Test B (χ^2^ (2) = 17.210; *P* < .001), the Regensburg Word Fluency Test (RWAT) K (χ^2^ (2) = 8.163; *P* = .017), RWAT S (χ^2^ (2) = 7.998; *P* = .018), RWAT Animals (χ^2^ (2) = 9.474; *P* = .009), RWAT Food (χ^2^ (2) = 16.271; *P* < .001) and the Digit Symbol Coding Test (χ^2^ (2) = 9.149; *P* = .010).

## Discussion

In the presented study, we evaluated the impact of a COVID-19 infection on structural alterations as well as neurocognitive functions. Acutely ill patients were examined using neuropsychological tests and MRI and compared to patients recovered from COVID-19 and a group of healthy controls.

We found significantly reduced grey matter in whole-brain analyses in patients with an acute COVID-19 infection in the left inferior frontal gyrus, the insular cortex and the basal ganglia compared to healthy controls. This decline could also be retraced in recovered patients compared to healthy controls in similar regions suggesting persistence of these changes over the period of acute illness. When compared to acutely ill, recovered patients showed an increase in grey matter volume in the cerebellum, hippocampus, parahippocampal gyrus in the temporal lobe, and fusiform gyrus, potentially representing recompensating changes in these areas over time. Nevertheless, in this comparison, areas of the basal ganglia and thalamus showed a secondary decrease in volume. In previous research, increases in grey matter volume in the olfactory cortex, insula, cingulate gyrus and hippocampus and the left rolandic operculum are described in recovered COVID-19 patients compared to healthy controls [7]. Additionally, Qin et al. [10] found a reduction of grey matter thickness/ grey matter volume in the left insula, the left hippocampus, the left superior temporal gyrus, left putamen and thalamus in patients with severe SARS-CoV-2 infection.

In addition to the whole-brain analysis, we also analyzed gray matter volume in ROI analyses. After adjustment of data to differences in intracranial volume (TIV) and the use of different MRI scanners, reductions were not only found in the areas already identified in the whole-brain analysis, but also in various areas distributed over the entire brain and both hemispheres especially in the group of acutely ill patients with a bihemispheric accentuation of the frontal lobe and the cerebellum. These findings are consistent with those of previous studies [41, 42].

Regarding white matter changes, in our analysis, patients with acute COVID-19 infection showed significantly lower tract volumes of the anterior thalamic radiation (ATR), connecting the thalamus with the frontal lobe [43]. In combination with the above mentioned grey matter effects with lower volumes in the left inferior frontal gyrus, the insular cortex and the basal ganglia, these changes in the frontal–basal ganglia–thalamic functional network [44, 45] could represent a specific correlate of COVID-19 infections in the CNS. This network has already been discussed in the context of a variety of diseases like Parkinson’s Disease, Tourette Syndrome, Obsessive Compulsive Disorder and dystonia, but has also been linked to motivation, sensorimotor adaption, learning, language processing and executive function [46, 47]. In recovered patients, apart from the right STR, these white matter changes, could already not be retraced any longer, suggesting reversibility. Also microstructural white matter changes represented by lower FA and higher MD in acutely ill patients seem to be reversible as nearly all of them couldn’t be retraced in the comparison of recovered cases and healthy controls any longer. Findings of reduced FA and increased MD have already been partially described in previous studies with respect to COVID-19 [8, 9], whereas one study reported conflictive findings [7].

Nevertheless, a detailed interpretation of the previously described structural findings on a neurofunctional base and the interaction of the several aspects like alterations in grey- and white matter as well as the time course of these factors is described in literature only partially. Especially the link of these changes on higher neurocognitive function has not been investigated yet in a comprehensive manner. In our study, both acute and recovered COVID-19 patients showed significant neurocognitive decline in several cognitive domains compared to healthy controls. These were primarily related to executive functions, language production, and attention. This is also consistent with results of previously published studies. Almeria et al. [14] showed that patients who reported neurocognitive deficits in the course of COVID-19 disease had these deficits in areas such as Digit Span Forward/ Backward, Trail Making Test B, or Lexical Fluency tests. A meta-analysis also found evidence of impairment in attention, executive functions, memory, language and visuospatial functions across multiple studies [48].

Interestingly, regarding grey matter changes, a strong unilaterality was found in our data for the left hemisphere within the whole-brain analyses. Moreover, this left hemispheric accentuated reduction of grey matter would be consistent with the deficits found in neuropsychological tests, especially in tests of language production. Acutely ill patients showed worse performance in the subtests of the RWAT in both the phonetic and semantic domains. Correspondingly, a reduction in the grey matter volume of the left inferior frontal gyrus, which contains Broca’s area and thus represents an important area for language processing [49], could be found. However, detailed ROI analyses showed effects for both hemispheres after adjustment for brain size and the use of different MRI scanners.

Additionally, ultrastructural changes, represented by significantly lower FA and higher MD, were found in long associative fiber tracts, primarily the Inferior fronto-occipital fasciculus (IFO) and the Middle longitudinal fasciculus (MdLF). These fiber tracts represent important structural connections within functional language networks [50]. The IFO, which was also found to have significantly lower volume in acute patients compared to controls on the right side, transfers information between the frontal cortex and the ipsilateral occipital, temporal and parietal lobe and was found to play, apart from language processing, a fundamental role in the network of attention, visual processing and executive control [51, 52]. The MdLF, fanning from the superior temporal gyrus to temporal and occipital areas, is suggested to mediate stimuli with respect to higher-order processing of acoustic information [53]. Also, lower normalized tract volumes were found for the Frontal Aslant tract (FAs), the Arcuate Fasciculus (AF) and the SLF 3 in acutely ill compared to recovered patients. A previous DTI study found specific patterns of white matter alterations in patients with severe SARS-CoV2 infection, also including ATR right, IFO right, ILF and the SLF2 left. Correspondingly to our findings, a decline of volume was found in infected patients, even if not all of these differences were significant in our study [10].

Correspondingly to the involved tracts, we found further neuropsychological deficits in the area of executive functions, which were reflected in the poorer performance of acutely ill patients in the Trail Making Test B and in the phonetic and semantic subtests of the RWAT. Even in recovered patients, a reduction of cognitive performance could still be retraced, as shown by significant differences in the RWAT Animals subtest, but also in a non-signifcant trend in RWAT subtests on letters B and K and the RWAT Food subtest. In accordance with these functional deficits, grey matter reduction was found in acutely ill patients in the left superior, middle and inferior frontal gyrus, as well as in the left superior and middle temporal gyrus. These regions have been found to be activated during the RWAT and the Trail Making Test in previous functional MRI studies [54, 55]. Regarding white matter, Loe et al. found an association between the FA of the cingulum, Forceps major, IFO, ILF, SLF, corticospinal tracts and partially the ATR and the strategy score, used to depict executive function, in children [56]. Many of these tracts also showed significant differences regarding normalized volumes or ultrastructural properties compared to controls or recovered in our study. Additionally, inter-hemispheric fibers like callosal or commissural fibers and the Forceps minor, have previously been discussed with respect to higher cognitive functions respectively cognitive decline [57–60]. Relations between executive functions and the frontal–basal ganglia–thalamic functional network have already been discussed above.

In this study, we present a multi-dimensional approach towards a comprehensive disease model of COVID-19 coupling grey- and white matter changes to functional neurocognitive evaluations and the temporal course over a SARS-CoV2 infection. However, regarding the selected methods and collected data, some limitations have to be taken into account. First, the significant age difference between the groups should be kept in mind with a higher mean age of the group of acutely ill patients compared to the other two study groups. This imbalance probably exists due to the lack of effective vaccination during the time of data collection between May 2020 and April 2021 just after the end of the first lockdown in Germany and age being considered as a risk factor for the need of hospitalization. In addition, volumes of CSF, grey matter, and white matter were found to differ between groups despite prior normalization. Strong correlations could be found between the volumes of the individual tissue types and the age of the subjects. To include this influence in the analyses, the respective patient age, TIV and disease severity were considered as a covariate in all grey matter analyses.

A major limitation is the use of two different MRI scanners which was necessary due to the high pandemic restrictions during the time of data acquisition between May 2020 and April 2021 including the time of the second German lockdown from January to May 2021. The study design with two MRI scanners at two healthcare centers was chosen in order not to put the cohort of acutely ill patients and healthcare professionals at risk due to clinically unnecessary transports of infectious patients between study sites as well as the cohort of healthy controls due to unnecessary exposure to SARS-CoV2. Nevertheless, these data from the relative beginning of the COVID-19 pandemic, when not the majority of people had been recovered from COVID-19 or vaccinated yet, could provide a valuable insight into the mechanisms of COVID-19 which can’t be retraced in these days any longer due to the high recovery and vaccination rate in nowadays populations. To minimize a possible influence of the use of different MRI scanners, which has previously been described for both gray and white matter [61], we examined a validation cohort on both scanners after the end of the pandemic. Based on these results, an individualized quotient was calculated for all examined tracts and brain areas, including possible scanner effects which should therefore reduce these artifacts between the examined patient groups. As based on the current literature, magnetic strength is not expected to affect results uniformly in different areas of the brain [62–64], individual quotients were calculated for each ROI and fiber tract instead of the use of one global parameter for all analyses. Alternative approaches based on COMBAT [65, 66] use more sophisticated models of region-specific harmonization between MRI scanners, but the usage of correctional factors has also been described in literature with acceptable results [67], even if less subjects were used for the validation cohort.

In conclusion, we found widespread changes in grey matter volume and white matter tracts in COVID-19 patients. Affected areas were mainly found in the frontal and temporal lobe, the basal ganglia, the cerebellum, and in fiber tracts connecting these areas with a left hemispheric accentuation. Overall, this revealed connections as previously described in networks of executive control and language as well as in the frontal–basal ganglia–thalamic functional network. These findings were also consistent with deficits found in the neuropsychological tests. White matter changes have been found to be regressive in recovered patients, whereas grey matter residua seem to outlast or at least recover more slowly. Recovered patients still showed declined scores in specific neuropsychological and neurocognitive tests.

Our data therefore provide a more differentiating point of view on the neurofunctional processes related to COVID-19, which could eventually lead towards a comprehensive disease model. In any case, comprehensive knowledge of the discussed disease-specific pathophysiological processes is necessary, also with respect to neurological and mental long-term impairments, often referred to as ‘Post-COVID’.

## Supporting information

S1 File The supporting information file contains S1 to S7 Tables and S1 to S7 Figs. (DOCX)

## Abbreviations

AC: Anterior Commissure
ACE2: Angiotensin-converting enzyme
AF: Arcuate Fasciculus
AR: Acoustic Radiation
ATR: Anterior Thalamic Radiation
CBD: dorsal Cingulum subsection
CBP: peri-genual Cingulum subsection
CBT: temporal Cingulum subsection
CNS: central nervous system
COVID-19: Coronavirus disease 2019
CSF: cortico spinal fluid
CST: Corticospinal Tract
DTI: Diffusion Tensor Imaging
FA: Fractional Anistropy
FAs: Frontal Aslant
FLAIR: FLuid Attenuated Inversion Recovery
FMA: Forceps Major
FMI: Forceps Minor
FoV: Field-of-view
FX: Fornix
ILF: Inferior Longitudinal Fasciculus
IFO: Inferior Fronto-Occipital Fasciculus
MCP: Middle Cerebellar Peduncle
MD: Mean Diffusivity
MdLF: Middle Longitudinal Fasciculus
MNI: Montreal Neurological Institute
MP-RAGE: Magnetization Prepared Rapid Acquisition with Gradient Echo
OR: Optic Radiation
ROI: Region Of Interest
RWAT: Regensburg Word Fluency Test
SARS-CoV2: severe acute respiratory syndrome coronavirus 2
SLF1/ 2/ 3: Superior Longitudinal Fasciculus 1/ 2/ 3; S-protein = spike-like protein
STR: Superior Thalamic Radiation
SWI: Susceptibility Weighted Image
TBSS: Tract-Based Spatial Statistics
TE: echo time
TFCE: Threshold-Free Cluster Enhancement
TIV: total intracranial volume
TR: repetition time
UF: Uncinate Fasciculus
VBM: voxel-based morphometry
VOF: Vertical Occipital Fasciculus
WAIS: Wechsler Adult Intelligence Scale
3D: three-dimensional
