# Supplementary material for: SARS-CoV2 evokes structural brain changes resulting in declined executive function
Source: PLoS One. 2024 Mar 12;19(3):e0298837. doi: 10.1371/journal.pone.0298837 (PMC10931481; doi:10.1371/journal.pone.0298837)
Supplement: S1 File — (DOCX) [file pone.0298837.s001.docx]

# Supporting Information

| **Region (mm3)** | **Left hemisphere** | | |  | **Right hemisphere** | | |
| --- | --- | --- | --- | --- | --- | --- | --- |
|  | **3T SIEMENS  Skyra Scanner** | **1.5T SIEMENS  Aera Scanner** | **Adjustment Quotient** |  | **3T SIEMENS  Skyra Scanner** | **1.5T SIEMENS  Aera Scanner** | **Adjustment Quotient** |
| Precentral gyrus | 11.124 | 9.860 | .886 |  | 1.107 | 8.699 | .861 |
| Superior frontal gyrus-dorsolateral | 14.438 | 13.903 | .963 |  | 14.645 | 14.179 | .968 |
| Middle frontal gyrus | 16.325 | 15.698 | .962 |  | 15.553 | 14.923 | .960 |
| Inferior frontal gyrus (opercular) | 3.999 | 3.789 | .947 |  | 5.080 | 4.703 | .926 |
| Inferior frontal gyrus (triangular) | 7.832 | 7.386 | .943 |  | 6.795 | 6.449 | .949 |
| Inferior frontal gyrus (orbitalis) | 2.611 | 2.492 | .954 |  | 2.634 | 2.524 | .958 |
| Rolandic operculum | 3.868 | 3.562 | .921 |  | 5.090 | 4.688 | .921 |
| Supplementary motor area | 7.051 | 6.689 | .949 |  | 7.242 | 6.727 | .929 |
| Olfactory cortex | 1.343 | 1.308 | .974 |  | 1.268 | 1.201 | .947 |
| Superior frontal gyrus-medial | 9.284 | 9.123 | .983 |  | 6.876 | 6.780 | .986 |
| Superior frontal gyrus-medial orbital | 2.818 | 2.753 | .977 |  | 3.259 | 3.129 | .960 |
| Gyrus rectus | 2.810 | 2.873 | 1.022 |  | 2.604 | 2.605 | 1.001 |
| Medial orbital gyrus | 2.082 | 2.114 | 1.016 |  | 2.292 | 2.316 | 1.011 |
| Anterior orbital gyrus | 2.130 | 2.051 | .963 |  | 2.643 | 2.607 | .986 |
| Posterior orbital gyrus | 2.569 | 2.443 | .951 |  | 2.300 | 2.177 | .946 |
| Lateral orbital gyrus | .888 | .874 | .984 |  | .678 | .662 | .977 |
| Insula | 8.498 | 7.921 | .932 |  | 9.050 | 8.341 | .922 |
| Anterior cingulate gyrus | 6.443 | 6.208 | .964 |  | 5.552 | 5.335 | .961 |
| Middle cingulate gyrus | 7.856 | 7.521 | .957 |  | 7.463 | 7.165 | .960 |
| Posterior cingulate gyrus | 1.621 | 1.545 | .953 |  | .880 | .847 | .963 |
| Hippocampus | 4.568 | 4.247 | .930 |  | 4.354 | 4.079 | .937 |
| Parahippocampal gyrus | 4.605 | 4.292 | .932 |  | 5.068 | 4.731 | .933 |
| Amygdala | 1.433 | 1.328 | .927 |  | 1.343 | 1.255 | .935 |
| Calcarine fissure and surrounding cortex | 7.285 | 6.400 | .878 |  | 5.533 | 4.741 | .857 |
| Cuneus | 4.902 | 4.431 | .904 |  | 4.776 | 4.329 | .906 |
| Lingual gyrus | 7.682 | 7.078 | .921 |  | 7.662 | 7.016 | .916 |
| Superior occipital gyrus | 3.392 | 3.128 | .922 |  | 3.948 | 3.638 | .921 |
| Middle occipital gyrus | 11.524 | 11.006 | .955 |  | 7.130 | 6.762 | .948 |
| Inferior occipital gyrus | 3.376 | 3.285 | .973 |  | 3.631 | 3.472 | .956 |
| Fusiform gyrus | 1.498 | 9.889 | .942 |  | 9.954 | 9.483 | .953 |
| Postcentral gyrus | 11.483 | 1.052 | .875 |  | 1.614 | 9.067 | .854 |
| Superior parietal gyrus | 6.878 | 6.587 | .958 |  | 7.197 | 6.818 | .947 |
| Inferior parietal gyrus | 8.990 | 8.597 | .956 |  | 5.009 | 4.727 | .944 |
| Supramarginal gyrus | 4.341 | 4.195 | .966 |  | 5.701 | 5.442 | .955 |
| Angular gyrus | 4.628 | 4.504 | .973 |  | 6.684 | 6.418 | .960 |
| Precuneus | 12.344 | 11.714 | .949 |  | 11.335 | 1.721 | .946 |
| Paracentral lobule | 3.442 | 2.790 | .811 |  | 2.409 | 1.976 | .820 |
| Caudate nucleus | 3.301 | 3.189 | .966 |  | 3.743 | 3.646 | .974 |
| Putamen | 4.558 | 4.263 | .935 |  | 4.725 | 4.379 | .927 |
| Pallidum | .223 | .205 | .917 |  | .522 | .466 | .893 |
| Thalamus | 4.133 | 3.502 | .847 |  | 4.055 | 3.363 | .829 |
| Heschls gyrus | .881 | .789 | .896 |  | .792 | .669 | .845 |
| Superior temporal gyrus | 1.137 | 9.498 | .937 |  | 1.828 | 1.070 | .930 |
| Temporal pole: superior temporal gyrus | 5.686 | 5.410 | .951 |  | 5.795 | 5.480 | .946 |
| Middle temporal gyrus | 18.980 | 18.246 | .961 |  | 17.146 | 16.395 | .956 |
| Temporal pole: middle temporal gyrus | 3.226 | 3.152 | .977 |  | 4.674 | 4.598 | .984 |
| Inferior temporal gyrus | 11.441 | 11.224 | .981 |  | 13.083 | 12.861 | .983 |
| Nucleus accumbens | .861 | .836 | .971 |  | .825 | .782 | .947 |
| Ventral tegmental area | < .001 | .003 | 9.740 |  | < .001 | .001 | 16.544 |
| Substantia nigra-pars compacta | < .001 | .002 | 7.348 |  | .003 | .003 | 1.122 |
| Substantia nigra-pars reticulata | .004 | .006 | 1.767 |  | .007 | .012 | 1.794 |
| Red nucleus | < .001 | .003 | 76.892 |  | .000 | .001 | 1.000 |
| Locus coeruleus | < .001 | < .001 | 1.000 |  | < .001 | < .001 | 1.000 |
| Raphe nucleus-dorsal | .019 | .012 | .633 |  |  |  |  |
| Raphe nucleus-medial | .002 | < .001 | .262 |  |  |  |  |
| Cerebellum | 43.485 | 42.232 | .971 |  | 43.727 | 42.296 | .967 |
| Vermis (bilateral) | 6.331 | 6.032 | .953 |  |  |  |  |

Supplementary Table 1: **Overview over mean gray matter volumes for the validational cohort used to correct for scanner artifacts.** Mean values are shown for each region of interest for both scanners used in this study as well as the calculated adjustment quotients.

| **Group** | **Cluster size** | **Cluster P_uncorr_** | **Cluster P_FDR-corr_** | **T** | **x** | **y** | **z** | **Brain regions (AAL3 Atlas)** |
| --- | --- | --- | --- | --- | --- | --- | --- | --- |
| Recovered patients > Acutely ill patients | 5135 | < .001 | .002 | 5.02 | -33 | -53 | -33 | Cerebellum 6 (L), Cerebellum Crus 1 (L), Fusiform gyrus (L), Cerebellum Crus 2 (L), Cerebellum 8 (L), Cerebellum 7b (L), Cerebellum 9 (L), Inferior temporal gyrus (L), Vermis 10, Parahippocampal gyrus (L), Cerebellum 4+5 (L), Vermis 9, Cerebellum 3 (L) |
|  | 6006 | < .001 | .002 | 4.20 | 27 | -51 | -56 | Cerebellum 8 (R), Cerebellum Crus 1 (R), Cerebellum 6 (R), Cerebellum Crus 2 (R), Cerebellum 7b (R), Cerebellum 9 (R), Cerebellum 10 (R), Vermis 9, Vermis 10, Vermis 8 |
|  |  |  |  |  |  |  |  |  |
| Acutely ill patients > Recovered patients | 2948 | .003 | .028 | 5.72 | 5 | -26 | 17 | Putamen (R), Thalamus medial pulvinar (R), Thalamus ventral lateral nucleus (R), Thalamus ventral posterolateral nucleus (R), Pallidum (R), Thalamus mediodorsal medial nucleus (R), Thalamus anterior pulvinar (R), Caudate (R), Thalamus lateral posterior nucleus (R), Thalamus lateral pulvinar (R), Thalamus intralaminar nucleus (R), Thalamus inferior pulvinar (R), Thalamus mediodorsal lateral nucleus (R), Thalamus ventral anterior nucleus (R), Thalamus medial geniculate nucleus (R), Lingual gyrus (R), Thalamus lateral geniculate nucleus (R), |
|  |  |  |  |  |  |  |  |  |
| Healthy Controls > Recovered patients | 3899 | .001 | .008 | 4.80 | -39 | 20 | 12 | Inferior frontal gyrus pars triangularis (L), Insula (L), Putamen (L), Inferior frontal gyrus pars opercularis (L), Inferior frontal gyrus pars orbitalis (L), Caudate (L) |
|  |  |  |  |  |  |  |  |  |
| Healthy Controls > Acutely ill patients | 3906 | .001 | .013 | 4.95 | -38 | 18 | 12 | Inferior frontal gyrus pars triangularis (L), Insula (L), Putamen (L), Inferior frontal gyrus pars opercularis (L), Inferior frontal gyrus pars orbitalis (L), Caudate (L), Nucleus accumbens (L) |

Supplementary Table 2: **Results of whole-brain grey matter analyses (T = 3) between the three study groups.** Anatomical labeling of the significant clusters was obtained using the Automated Anatomical Labeling 3 (AAL3) atlas implemented in SPM12. Abbreviations: B = bilateral, L = left, R = right.

| **Region (mm3)** | **Left hemisphere** | | | | |  | **Right hemisphere** | | | | |
| --- | --- | --- | --- | --- | --- | --- | --- | --- | --- | --- | --- |
|  | **Acutely ill  COVID-19 patients** | **Recovered  COVID-19 patients** | **Healthy  controls** | **F** | **p value** |  | **Acutely ill  COVID-19 patients** | **Recovered  COVID-19 patients** | **Healthy  controls** | **F** | **p value** |
| Precentral gyrus | 10761.387 | 11547.887 | 11858.690 | .151 | .937 |  | 9737.440 | 10168.138 | 10591.639 | .635 | .781 |
| Superior frontal gyrus-dorsolateral | 15331.257 | 17359.905 | 17725.475 | .397 | .851 |  | 15365.033 | 17678.269 | 17563.030 | .713 | .747 |
| Middle frontal gyrus | 15317.664 | 18204.292 | 18022.517 | 2.773 | .305 |  | 1620.789 | 18504.655 | 1941.885 | 2.405 | .336 |
| Inferior frontal gyrus (opercular) | 352.144 | 4264.393 | 4339.543 | 3.129 | .281 |  | 4774.615 | 5785.000 | 5712.500 | 2.044 | .386 |
| Inferior frontal gyrus (triangular) | 7486.946 | 9209.064 | 9979.157 | 8.217 | .032 * |  | 6235.184 | 768.051 | 798.460 | 5.148 | .102 |
| Inferior frontal gyrus (orbitalis) | 2286.526 | 2801.555 | 2988.508 | 6.684 | .049 * |  | 2577.781 | 3075.741 | 3313.042 | 4.470 | .134 |
| Rolandic operculum | 382.088 | 4589.077 | 4603.034 | 1.965 | .393 |  | 4811.311 | 5756.847 | 5878.605 | 3.100 | .281 |
| Supplementary motor area | 6857.708 | 8015.245 | 7683.102 | 1.945 | .393 |  | 7414.172 | 7807.411 | 7866.981 | 1.107 | .620 |
| Olfactory cortex | 1479.925 | 1573.444 | 157.898 | .021 | .988 |  | 1394.465 | 1391.518 | 1406.048 | 1.138 | .620 |
| Superior frontal gyrus-medial | 9496.449 | 10755.281 | 10616.122 | .285 | .889 |  | 7282.132 | 7955.746 | 8112.827 | .267 | .889 |
| Superior frontal gyrus-medial orbital | 2869.670 | 3185.777 | 324.486 | .029 | .988 |  | 3495.227 | 4009.851 | 414.503 | 1.139 | .620 |
| Gyrus rectus | 3041.430 | 3204.672 | 3218.362 | .293 | .889 |  | 2827.152 | 3039.783 | 3142.858 | .210 | .919 |
| Medial orbital gyrus | 2252.282 | 2409.778 | 2415.483 | .009 | .991 |  | 2627.083 | 2751.718 | 2855.766 | .556 | .782 |
| Anterior orbital gyrus | 2188.797 | 2451.077 | 2521.344 | .811 | .713 |  | 2977.210 | 3377.671 | 3343.226 | .528 | .794 |
| Posterior orbital gyrus | 2453.436 | 2737.616 | 2951.075 | 2.619 | .326 |  | 218.297 | 2491.078 | 2638.554 | 2.883 | .301 |
| Lateral orbital gyrus | 862.427 | 995.169 | 1051.804 | 2.502 | .336 |  | 749.056 | 799.468 | 84.700 | .306 | .889 |
| Insula | 8532.326 | 9345.805 | 10032.743 | 2.869 | .301 |  | 8529.510 | 9635.152 | 10306.473 | 2.103 | .383 |
| Anterior cingulate gyrus | 6488.928 | 7473.493 | 7391.259 | .324 | .889 |  | 5741.452 | 6402.105 | 6346.302 | .157 | .937 |
| Middle cingulate gyrus | 8224.164 | 9401.102 | 9417.111 | .617 | .781 |  | 8234.625 | 919.378 | 9342.783 | .265 | .889 |
| Posterior cingulate gyrus | 169.367 | 1799.136 | 1841.120 | .124 | .943 |  | 1106.401 | 1102.052 | 1184.906 | 1.661 | .443 |
| Hippocampus | 5004.922 | 5622.463 | 5299.684 | 2.428 | .336 |  | 4796.489 | 5383.249 | 5041.573 | 2.231 | .371 |
| Parahippocampal gyrus | 5096.140 | 5587.079 | 5502.958 | .967 | .648 |  | 5917.093 | 6453.942 | 615.555 | 3.126 | .281 |
| Amygdala | 1551.917 | 1679.556 | 1661.717 | .591 | .781 |  | 1503.179 | 160.659 | 1572.027 | .709 | .747 |
| Calcarine fissure and surrounding cortex | 7275.163 | 7736.526 | 8226.078 | 1.505 | .490 |  | 651.163 | 667.811 | 6687.408 | 1.079 | .620 |
| Cuneus | 524.116 | 5767.511 | 5933.609 | .168 | .937 |  | 5446.737 | 5744.537 | 5569.792 | 1.475 | .495 |
| Lingual gyrus | 7667.162 | 8467.978 | 9073.669 | 3.684 | .220 |  | 8272.146 | 9316.528 | 9173.931 | 1.170 | .620 |
| Superior occipital gyrus | 3827.478 | 4103.319 | 4489.662 | 1.677 | .443 |  | 4493.549 | 460.540 | 4776.993 | .800 | .713 |
| Middle occipital gyrus | 11344.720 | 12522.617 | 13042.734 | .645 | .781 |  | 7568.766 | 8369.662 | 8569.370 | .095 | .953 |
| Inferior occipital gyrus | 295.079 | 3367.669 | 3486.874 | .715 | .747 |  | 3685.955 | 4266.120 | 4316.774 | .961 | .648 |
| Fusiform gyrus | 10442.198 | 12146.172 | 11831.910 | 5.809 | .067 |  | 10724.059 | 12231.368 | 12356.257 | 2.456 | .336 |
| Postcentral gyrus | 11087.486 | 12288.461 | 12837.467 | .327 | .889 |  | 1032.091 | 11641.059 | 11979.407 | .468 | .824 |
| Superior parietal gyrus | 7049.322 | 7539.151 | 7745.400 | .137 | .940 |  | 7271.153 | 8095.262 | 8234.715 | .439 | .836 |
| Inferior parietal gyrus | 8625.559 | 10183.929 | 10226.490 | .985 | .648 |  | 5492.140 | 6604.087 | 6416.260 | 1.789 | .434 |
| Supramarginal gyrus | 4579.290 | 5393.977 | 5422.819 | 1.730 | .439 |  | 6503.578 | 7339.690 | 7459.892 | .408 | .851 |
| Angular gyrus | 4448.056 | 5246.729 | 4982.655 | 2.342 | .345 |  | 6837.863 | 8071.466 | 7791.067 | 1.748 | .439 |
| Precuneus | 13837.913 | 14959.658 | 15226.635 | .047 | .981 |  | 12489.527 | 13638.304 | 13682.926 | .466 | .824 |
| Paracentral lobule | 3676.171 | 3736.253 | 3894.001 | 1.126 | .620 |  | 2552.059 | 2516.371 | 2658.343 | 2.134 | .383 |
| Caudate nucleus | 3294.257 | 3473.322 | 3666.425 | .925 | .660 |  | 4067.892 | 4171.033 | 4395.952 | .860 | .693 |
| Putamen | 4892.436 | 4635.255 | 4926.821 | 7.001 | .045 * |  | 5238.722 | 4891.694 | 5134.062 | 6.347 | .053 |
| Pallidum | 29.065 | 224.505 | 211.208 | 2.717 | .309 |  | 588.180 | 508.366 | 526.172 | 4.065 | .170 |
| Thalamus | 4133.461 | 3373.552 | 3349.276 | 3.620 | .220 |  | 4158.688 | 3137.448 | 2983.066 | 7.691 | .032 * |
| Heschls gyrus | 894.193 | 1084.593 | 1123.692 | 1.103 | .620 |  | 812.519 | 897.385 | 1009.726 | 2.080 | .383 |
| Superior temporal gyrus | 10244.543 | 11895.329 | 11826.114 | .600 | .781 |  | 10456.308 | 11799.541 | 11978.838 | .297 | .889 |
| Temporal pole: superior temporal gyrus | 5756.846 | 6464.338 | 6336.765 | 1.079 | .620 |  | 5814.286 | 673.714 | 6765.326 | 2.125 | .383 |
| Middle temporal gyrus | 17892.693 | 21566.491 | 2091.501 | 4.648 | .134 |  | 17211.171 | 20246.019 | 19832.652 | 2.830 | .301 |
| Temporal pole: middle temporal gyrus | 3579.988 | 3917.403 | 3815.881 | 1.054 | .620 |  | 4979.188 | 5602.008 | 5633.046 | 1.523 | .490 |
| Inferior temporal gyrus | 11254.990 | 13108.068 | 12912.100 | 3.455 | .240 |  | 13723.389 | 15868.414 | 15952.264 | 2.945 | .301 |
| Nucleus accumbens | 953.820 | 1061.526 | 1088.356 | .573 | .781 |  | 941.596 | 1024.016 | 1044.907 | .099 | .953 |
| Ventral tegmental area | .382 | 5.036 | 4.258 | 12.316 | .006 ** |  | .159 | 2.839 | 2.249 | 7.701 | .032 * |
| Substantia nigra-pars compacta | 1.159 | .502 | .691 | .570 | .781 |  | 3.023 | 1.204 | 1.316 | 1.963 | .393 |
| Substantia nigra-pars reticulata | 6.731 | 4.064 | 5.530 | 1.057 | .620 |  | 6.409 | 6.758 | 5.324 | .179 | .937 |
| Red nucleus | .082 | .739 | .937 | 4.432 | .134 |  | .112 | .210 | .359 | 1.656 | .443 |
| Locus coeruleus | .033 | .015 | .040 | .225 | .915 |  | .676 | .032 | .125 | 1.887 | .406 |
| Raphe nucleus-dorsal | 47.684 | 25.910 | 26.580 | 11.435 | .006 ** |  |  |  |  |  |  |
| Raphe nucleus-medial | 7.893 | 3.030 | 4.041 | 2.577 | .327 |  |  |  |  |  |  |
| Cerebellum | 43604.933 | 53544.512 | 5339.615 | 4.484 | .134 |  | 46167.153 | 57452.381 | 56715.337 | 6.242 | .053 |
| Vermis (bilateral) | 7159.311 | 7602.180 | 7766.151 | .082 | .956 |  |  |  |  |  |  |

Supplementary Table 3: **Overview of all results of the grey matter ROI analysis.** Individual subject data was normalized using total intracranial volume before analysis, subject age was used as a covariate, and p-values of individual analyses were adjusted using Benjamini-Hochberg correction for multiple testing. Brain areas for which no clear assignment to a hemisphere is possible are shown on the left side of the table. Statistically significant results were marked with * for P < .05, ** for P < .01 and *** for P < .001.

| **Region (mm3)** | **Left hemisphere** | | | | |  | **Right hemisphere** | | | | |
| --- | --- | --- | --- | --- | --- | --- | --- | --- | --- | --- | --- |
|  | **Acutely ill  COVID-19 patients** | **Recovered  COVID-19 patients** | **Healthy  controls** | **F** | **p value** |  | **Acutely ill  COVID-19 patients** | **Recovered  COVID-19 patients** | **Healthy  controls** | **F** | **p value** |
| Precentral gyrus | 1229.084 | 14836.096 | 15106.811 | 4.342 | .055 |  | 1080.139 | 13056.752 | 13465.032 | 4.228 | .055 |
| Superior frontal gyrus-dorsolateral | 19004.728 | 22232.879 | 22551.586 | 3.339 | .086 |  | 19216.891 | 22646.892 | 22345.890 | 3.063 | .101 |
| Middle frontal gyrus | 18921.008 | 23265.475 | 22951.823 | 6.068 | .032 * |  | 19932.990 | 23696.374 | 24746.113 | 4.979 | .042 * |
| Inferior frontal gyrus (opercular) | 4317.456 | 5457.800 | 5507.781 | 6.698 | .025 * |  | 5692.085 | 7434.155 | 724.438 | 6.811 | .025 * |
| Inferior frontal gyrus (triangular) | 9121.826 | 11817.568 | 12675.996 | 9.816 | .022 * |  | 7614.414 | 9832.335 | 10186.084 | 8.506 | .022 * |
| Inferior frontal gyrus (orbitalis) | 281.113 | 3602.148 | 3784.429 | 8.730 | .022 * |  | 317.236 | 3942.705 | 4214.652 | 7.538 | .022 * |
| Rolandic operculum | 4548.973 | 5861.149 | 5853.177 | 7.628 | .022 * |  | 5748.266 | 7372.352 | 7455.224 | 7.635 | .022 * |
| Supplementary motor area | 8382.216 | 10265.218 | 9761.822 | 4.529 | .053 |  | 884.612 | 9987.972 | 9997.996 | 2.269 | .171 |
| Olfactory cortex | 1854.384 | 2011.010 | 1993.660 | .952 | .476 |  | 1698.920 | 1777.123 | 1791.178 | .394 | .734 |
| Superior frontal gyrus-medial | 12037.725 | 13762.664 | 13536.149 | 1.904 | .224 |  | 9249.881 | 10186.277 | 10324.859 | 1.558 | .291 |
| Superior frontal gyrus-medial orbital | 3625.300 | 4074.576 | 4112.793 | 1.634 | .281 |  | 4325.818 | 5098.849 | 5251.909 | 5.171 | .042 * |
| Gyrus rectus | 4025.230 | 4088.894 | 4084.599 | .029 | .971 |  | 3649.170 | 3862.339 | 399.382 | .745 | .550 |
| Medial orbital gyrus | 2945.436 | 3086.804 | 3078.381 | .291 | .792 |  | 3398.456 | 3524.148 | 3636.258 | .560 | .645 |
| Anterior orbital gyrus | 2713.285 | 3137.803 | 3206.320 | 3.385 | .084 |  | 3776.264 | 4327.928 | 4257.642 | 2.279 | .171 |
| Posterior orbital gyrus | 2998.971 | 3509.926 | 374.771 | 4.981 | .042 * |  | 2652.714 | 3193.637 | 3346.490 | 5.746 | .038 * |
| Lateral orbital gyrus | 109.219 | 1277.850 | 1338.155 | 3.751 | .070 |  | 937.136 | 1026.459 | 1074.196 | 1.245 | .368 |
| Insula | 10276.975 | 11982.154 | 12776.192 | 4.519 | .053 |  | 10182.012 | 12341.770 | 13109.810 | 5.633 | .040 * |
| Anterior cingulate gyrus | 8052.343 | 9544.000 | 941.833 | 3.280 | .089 |  | 7097.144 | 8165.000 | 809.833 | 2.653 | .131 |
| Middle cingulate gyrus | 10129.237 | 12025.133 | 11966.378 | 4.242 | .055 |  | 1016.093 | 11743.241 | 11863.199 | 3.654 | .073 |
| Posterior cingulate gyrus | 2073.691 | 2299.294 | 2334.232 | 1.405 | .325 |  | 1375.986 | 1413.868 | 1499.920 | .677 | .582 |
| Hippocampus | 6016.876 | 7217.448 | 677.804 | 3.460 | .082 |  | 5826.213 | 6927.156 | 6434.862 | 2.678 | .131 |
| Parahippocampal gyrus | 6121.002 | 7153.938 | 701.550 | 3.523 | .079 |  | 712.050 | 8252.479 | 783.005 | 3.413 | .084 |
| Amygdala | 1857.433 | 2149.380 | 2117.306 | 3.101 | .101 |  | 181.757 | 2045.162 | 2002.053 | 2.651 | .131 |
| Calcarine fissure and surrounding cortex | 8218.236 | 9893.840 | 10479.796 | 6.302 | .028 * |  | 7166.977 | 8555.001 | 8519.068 | 3.696 | .072 |
| Cuneus | 609.597 | 7386.445 | 7552.025 | 4.413 | .055 |  | 6366.874 | 738.778 | 7068.136 | 2.220 | .172 |
| Lingual gyrus | 9089.643 | 10824.393 | 11547.417 | 6.943 | .025 * |  | 974.187 | 11945.866 | 11683.952 | 5.351 | .041 * |
| Superior occipital gyrus | 4536.260 | 5254.750 | 5712.096 | 4.353 | .055 |  | 5316.841 | 5926.510 | 6063.042 | 1.599 | .283 |
| Middle occipital gyrus | 13947.746 | 16064.452 | 16568.699 | 3.116 | .101 |  | 9236.121 | 10709.209 | 10875.773 | 2.918 | .111 |
| Inferior occipital gyrus | 3695.368 | 4306.315 | 4403.056 | 3.595 | .076 |  | 4502.403 | 5435.629 | 5468.450 | 5.142 | .042 * |
| Fusiform gyrus | 12686.239 | 15519.352 | 15035.675 | 6.646 | .025 * |  | 13161.995 | 15662.239 | 15686.315 | 5.072 | .042 * |
| Postcentral gyrus | 12532.061 | 15819.666 | 16277.821 | 5.332 | .041 * |  | 11357.512 | 14942.094 | 15201.104 | 8.207 | .022 * |
| Superior parietal gyrus | 8709.261 | 9651.945 | 9866.182 | 1.617 | .282 |  | 8882.421 | 10386.865 | 10504.376 | 2.724 | .128 |
| Inferior parietal gyrus | 10668.537 | 13057.811 | 12953.791 | 4.188 | .055 |  | 6686.553 | 849.281 | 8179.727 | 4.881 | .043 * |
| Supramarginal gyrus | 5726.145 | 6896.901 | 6876.067 | 4.240 | .055 |  | 8036.461 | 9405.103 | 9491.975 | 3.034 | .102 |
| Angular gyrus | 5564.431 | 6728.037 | 6319.852 | 4.026 | .058 |  | 8468.884 | 10348.482 | 9874.092 | 4.301 | .055 |
| Precuneus | 16934.867 | 19195.020 | 1929.112 | 2.234 | .172 |  | 15235.847 | 17479.091 | 17391.203 | 2.883 | .113 |
| Paracentral lobule | 3838.173 | 4803.884 | 4954.654 | 4.886 | .043 * |  | 2686.397 | 3223.622 | 3378.456 | 4.112 | .057 |
| Caudate nucleus | 4098.023 | 4456.499 | 4674.635 | 1.421 | .325 |  | 5082.660 | 5339.982 | 5609.216 | .867 | .500 |
| Putamen | 5904.541 | 593.158 | 6265.371 | .431 | .717 |  | 6277.901 | 626.724 | 6539.642 | .212 | .848 |
| Pallidum | 337.091 | 284.537 | 269.734 | 1.873 | .228 |  | 671.889 | 648.717 | 67.197 | .137 | .905 |
| Thalamus | 455.303 | 4395.136 | 4284.829 | .122 | .910 |  | 4467.844 | 4096.264 | 3819.546 | .827 | .514 |
| Heschls gyrus | 1035.675 | 1396.359 | 1427.261 | 6.671 | .025 * |  | 894.847 | 1154.549 | 1277.264 | 7.290 | .024 * |
| Superior temporal gyrus | 12436.094 | 15226.550 | 14997.157 | 4.598 | .053 |  | 12605.219 | 15103.888 | 15196.232 | 4.098 | .057 |
| Temporal pole: superior temporal gyrus | 7069.837 | 826.752 | 8051.992 | 3.060 | .101 |  | 7092.052 | 8617.614 | 8591.763 | 5.071 | .042 * |
| Middle temporal gyrus | 22265.446 | 27621.084 | 26563.066 | 5.519 | .041 * |  | 21235.222 | 25883.371 | 25194.026 | 5.306 | .041 * |
| Temporal pole: middle temporal gyrus | 4508.892 | 5015.179 | 4858.298 | 1.401 | .325 |  | 6308.827 | 7177.133 | 7161.914 | 2.451 | .154 |
| Inferior temporal gyrus | 14231.498 | 16777.680 | 16389.928 | 3.889 | .064 |  | 17365.477 | 20286.627 | 20233.061 | 4.217 | .055 |
| Nucleus accumbens | 1198.501 | 1358.325 | 1385.339 | 2.320 | .168 |  | 1154.266 | 131.629 | 133.666 | 2.258 | .171 |
| Ventral tegmental area | 5.088 | 6.185 | 5.157 | .065 | .946 |  | 3.752 | 3.519 | 2.750 | .077 | .943 |
| Substantia nigra-pars compacta | 11.373 | .631 | .900 | 8.546 | .022 * |  | 4.222 | 1.565 | 1.661 | 2.392 | .160 |
| Substantia nigra-pars reticulata | 15.267 | 5.154 | 7.462 | 4.041 | .058 |  | 15.976 | 8.722 | 6.875 | .868 | .500 |
| Red nucleus | .088 | .891 | 1.152 | 2.012 | .206 |  | .164 | .264 | .419 | .463 | .702 |
| Locus coeruleus | .048 | .018 | .057 | .303 | .790 |  | .968 | .038 | .159 | 1.219 | .373 |
| Raphe nucleus-dorsal | 39.462 | 33.911 | 34.433 | .388 | .734 |  |  |  |  |  |  |
| Raphe nucleus-medial | 2.678 | 3.991 | 5.134 | .878 | .500 |  |  |  |  |  |  |
| Cerebellum | 54873.557 | 68683.583 | 67924.111 | 5.453 | .041 * |  | 57834.356 | 73646.455 | 72145.876 | 6.522 | .025 * |
| Vermis (bilateral) | 8847.149 | 9733.222 | 9864.775 | 1.276 | .361 |  |  |  |  |  |  |

Supplementary Table 4: **Overview of all results of the grey matter ROI analysis after adjustment of data in order to minimize scanner effects based on the validational cohort.** Individual subject data was normalized using total intracranial volume and adjusted using the calculated scanner quotient before analysis, subject age was used as a covariate, and p-values of individual analyses were adjusted using Benjamini-Hochberg correction for multiple testing. Brain areas for which no clear assignment to a hemisphere is possible are shown on the left side of the table. Statistically significant results were marked with * for P < .05, ** for P < .01 and *** for P < .001.

| **Region (mm3)** | **Left hemisphere** |  |  |  |  |  | **Right hemisphere** | |  |  |  |
| --- | --- | --- | --- | --- | --- | --- | --- | --- | --- | --- | --- |
|  | **Acutely ill**  **COVID-19**  **patients** | **Recovered**  **COVID-19**  **patients** | **Healthy**  **controls** | **F** | **p value** |  | **Acutely ill**  **COVID-19**  **patients** | **Recovered**  **COVID-19**  **patients** | **Healthy**  **controls** | **F** | **p value** |
| Fronto-marginal gyrus (of Wernicke) and sulcus | 2.0926 | 2.3375 | 2.3883 | 14.284 | < .001 *** |  | 2.1550 | 2.4330 | 2.4542 | 18.253 | < .001 *** |
| Inferior occipital gyrus (O3) and sulcus | 2.1034 | 2.3395 | 2.3017 | 9.940 | < .001 *** |  | 2.1697 | 2.3485 | 2.3908 | 6.779 | .003 ** |
| Paracentral lobule and sulcus | 1.4648 | 1.8380 | 1.9383 | 16.604 | < .001 *** |  | 1.4657 | 1.8100 | 1.9833 | 13.271 | < .001 *** |
| Subcentral gyrus (central operculum) and sulci | 2.2438 | 2.4750 | 2.6100 | 15.815 | < .001 *** |  | 2.1998 | 2.4685 | 2.5867 | 16.712 | < .001 *** |
| Transverse frontopolar gyri and sulci | 2.1794 | 2.4770 | 2.5408 | 12.171 | < .001 *** |  | 2.1475 | 2.4820 | 2.5658 | 17.319 | < .001 *** |
| Anterior part of the cingulate gyrus and sulcus (ACC) | 2.4582 | 2.5960 | 2.6767 | 6.271 | .004 ** |  | 2.4486 | 2.6200 | 2.7033 | 7.986 | .001 ** |
| Middle-anterior part of the cingulate gyrus and sulcus (aMCC) | 2.4502 | 2.6430 | 2.7275 | 7.871 | .002 ** |  | 2.4510 | 2.6795 | 2.8000 | 12.434 | < .001 *** |
| Middle-posterior part of the cingulate gyrus and sulcus (pMCC) | 2.1920 | 2.4215 | 2.5158 | 16.921 | < .001 *** |  | 2.2421 | 2.4770 | 2.5200 | 15.500 | < .001 *** |
| Posterior-dorsal part of the cingulate gyrus (dPCC) | 2.5735 | 2.7030 | 2.8525 | 1.263 | < .001 *** |  | 2.5534 | 2.6575 | 2.7633 | 6.029 | .006 ** |
| Posterior-ventral part of the cingulate gyrus (vPCC. isthmus of the cingulate gyrus) | 2.0580 | 2.3885 | 2.3858 | 7.627 | .002 ** |  | 2.1679 | 2.4630 | 2.5125 | 7.968 | .001 ** |
| Cuneus (O6) | 1.4910 | 1.7370 | 1.7808 | 15.338 | < .001 *** |  | 1.5566 | 1.7460 | 1.8175 | 11.177 | < .001 *** |
| Opercular part of the inferior frontal gyrus | 2.3363 | 2.6720 | 2.7400 | 19.897 | < .001 *** |  | 2.3323 | 2.7070 | 2.7833 | 26.427 | < .001 *** |
| Orbital part of the inferior frontal gyrus | 2.3146 | 2.7230 | 2.7725 | 19.303 | < .001 *** |  | 2.3148 | 2.6225 | 2.6842 | 9.756 | < .001 *** |
| Triangular part of the inferior frontal gyrus | 2.1701 | 2.5155 | 2.5633 | 2.804 | < .001 *** |  | 2.2612 | 2.5250 | 2.6058 | 14.627 | < .001 *** |
| Middle frontal gyrus (F2) | 2.2160 | 2.5380 | 2.5900 | 18.475 | < .001 *** |  | 2.2017 | 2.5195 | 2.5583 | 17.761 | < .001 *** |
| Superior frontal gyrus (F1) | 2.3721 | 2.6550 | 2.7767 | 13.137 | < .001 *** |  | 2.3893 | 2.6995 | 2.7792 | 14.442 | < .001 *** |
| Long insular gyrus and central sulcus of the insula | 2.9039 | 2.9945 | 3.0025 | .214 | .813 |  | 2.7474 | 2.9245 | 3.0642 | 2.433 | .108 |
| Short insular gyri | 3.6279 | 3.4560 | 3.4683 | .774 | .481 |  | 3.3752 | 3.3010 | 3.4950 | 1.171 | .334 |
| Middle occipital gyrus (O2. lateral occipital gyrus) | 2.1557 | 2.3665 | 2.4050 | 13.219 | < .001 *** |  | 2.1729 | 2.3595 | 2.4075 | 8.382 | .001 ** |
| Superior occipital gyrus (O1) | 1.7881 | 2.0175 | 2.0767 | 11.173 | < .001 *** |  | 1.8558 | 2.0360 | 2.1117 | 8.001 | .001 ** |
| Lateral occipito-temporal gyrus (fusiform gyrus. O4-T4) | 2.2306 | 2.5045 | 2.5967 | 16.714 | < .001 *** |  | 2.2077 | 2.4570 | 2.5300 | 12.020 | < .001 *** |
| Lingual gyrus. ligual part of the medial occipito-temporal gyrus. (O5) | 1.6061 | 1.8675 | 1.9292 | 2.605 | < .001 *** |  | 1.6991 | 1.9580 | 2.0183 | 24.821 | < .001 *** |
| Parahippocampal gyrus. parahippocampal part of the medial occipito-temporal gyrus. (T5) | 2.6495 | 2.9800 | 3.0308 | 19.199 | < .001 *** |  | 2.8365 | 3.0980 | 3.1833 | 11.278 | < .001 *** |
| Orbital gyri | 2.4431 | 2.6515 | 2.6917 | 9.087 | < .001 *** |  | 2.4773 | 2.6955 | 2.7717 | 9.814 | < .001 *** |
| Angular gyrus | 2.2872 | 2.5035 | 2.5600 | 1.035 | < .001 *** |  | 2.2543 | 2.5215 | 2.5717 | 16.056 | < .001 *** |
| Supramarginal gyrus | 2.3257 | 2.5445 | 2.6150 | 11.946 | < .001 *** |  | 2.2834 | 2.5455 | 2.6492 | 24.860 | < .001 *** |
| Superior parietal lobule (lateral part of P1) | 2.0798 | 2.3125 | 2.3592 | 18.466 | < .001 *** |  | 2.0580 | 2.2730 | 2.3225 | 11.065 | < .001 *** |
| Postcentral gyrus | 1.5638 | 1.8910 | 1.9725 | 16.893 | < .001 *** |  | 1.4619 | 1.8350 | 1.9292 | 18.600 | < .001 *** |
| Precentral gyrus | 1.7467 | 2.0975 | 2.2650 | 14.827 | < .001 *** |  | 1.6332 | 2.0205 | 2.2375 | 17.024 | < .001 *** |
| Precuneus (medial part of P1) | 2.2070 | 2.4215 | 2.4883 | 14.670 | < .001 *** |  | 2.2080 | 2.4020 | 2.4942 | 15.889 | < .001 *** |
| Straight gyrus. Gyrus rectus | 2.2537 | 2.4340 | 2.4750 | 11.440 | < .001 *** |  | 2.3502 | 2.5510 | 2.6225 | 1.417 | < .001 *** |
| Subcallosal area. subcallosal gyrus | 2.6105 | 2.6955 | 2.7958 | 1.201 | .327 |  | 2.9266 | 3.1470 | 3.1583 | 2.419 | .109 |
| Anterior transverse temporal gyrus (of Heschl) | 1.7322 | 2.2545 | 2.2442 | 14.089 | < .001 *** |  | 1.8447 | 2.2040 | 2.4075 | 17.385 | < .001 *** |
| Lateral aspect of the superior temporal gyrus | 2.6161 | 2.8705 | 2.8908 | 8.118 | .001 ** |  | 2.6078 | 2.8895 | 2.9283 | 8.831 | < .001 *** |
| Planum polare of the superior temporal gyrus | 3.1134 | 3.4320 | 3.3050 | 2.686 | .088 |  | 3.2409 | 3.3450 | 3.4792 | 2.478 | .105 |
| Planum temporale or temporal plane of the superior temporal gyrus | 2.1743 | 2.5030 | 2.5575 | 13.341 | < .001 *** |  | 2.0867 | 2.4555 | 2.5558 | 18.782 | < .001 *** |
| Inferior temporal gyrus (T3) | 2.5605 | 2.7300 | 2.7633 | 11.880 | < .001 *** |  | 2.5795 | 2.7800 | 2.7975 | 1.259 | < .001 *** |
| Middle temporal gyrus (T2) | 2.5755 | 2.8000 | 2.8533 | 14.126 | < .001 *** |  | 2.6441 | 2.8475 | 2.9333 | 12.552 | < .001 *** |
| Horizontal ramus of the anterior segment of the lateral sulcus (or ﬁssure) | 2.0520 | 2.4745 | 2.5592 | 17.180 | < .001 *** |  | 2.0801 | 2.4815 | 2.5717 | 26.458 | < .001 *** |
| Vertical ramus of the anterior segment of the lateral sulcus (or ﬁssure) | 2.1159 | 2.5810 | 2.6350 | 21.581 | < .001 *** |  | 2.2019 | 2.5930 | 2.6500 | 19.680 | < .001 *** |
| Posterior ramus (or segment) of the lateral sulcus (or ﬁssure) | 1.9282 | 2.2920 | 2.4467 | 21.691 | < .001 *** |  | 2.1622 | 2.4315 | 2.5758 | 14.873 | < .001 *** |
| Occipital pole | 1.6315 | 1.7470 | 1.7933 | 3.486 | .044 * |  | 1.6312 | 1.7850 | 1.8283 | 8.131 | .001 ** |
| Temporal pole | 3.1275 | 3.3255 | 3.2800 | 3.494 | .044 * |  | 3.0286 | 3.2930 | 3.3192 | 6.363 | .005 ** |
| Calcarine sulcus | 1.5807 | 1.8040 | 1.8542 | 15.412 | < .001 *** |  | 1.5624 | 1.8370 | 1.8967 | 24.101 | < .001 *** |
| Central sulcus (Rolando's ﬁssure) | 1.1685 | 1.4770 | 1.5400 | 16.012 | < .001 *** |  | 1.1401 | 1.4600 | 1.5392 | 2.833 | < .001 *** |
| Marginal branch (or part) of the cingulate sulcus | 1.9825 | 2.2385 | 2.2800 | 17.821 | < .001 *** |  | 1.9452 | 2.2305 | 2.2825 | 19.339 | < .001 *** |
| Anterior segment of the circular sulcus of the insula | 2.8955 | 3.0045 | 2.9192 | .892 | .432 |  | 2.8690 | 2.8875 | 2.9608 | .592 | .565 |
| Inferior segment of the circular sulcus of the insula | 2.8173 | 2.9595 | 2.9475 | .609 | .560 |  | 2.8159 | 3.0230 | 2.8800 | 1.869 | .176 |
| Superior segment of the circular sulcus of the insula | 2.5505 | 2.7305 | 2.8375 | 7.804 | .002 ** |  | 2.6721 | 2.8135 | 2.9300 | 6.609 | .004 ** |
| Anterior transverse collateral sulcus | 2.4939 | 2.6840 | 2.7392 | 9.120 | < .001 *** |  | 2.4368 | 2.6465 | 2.6758 | 8.463 | .001 ** |
| Posterior transverse collateral sulcus | 1.7820 | 2.0190 | 2.0450 | 11.558 | < .001 *** |  | 1.7137 | 1.9790 | 2.0458 | 1.750 | < .001 *** |
| Inferior frontal sulcus | 1.9392 | 2.3325 | 2.4100 | 29.180 | < .001 *** |  | 1.9619 | 2.3205 | 2.3917 | 26.519 | < .001 *** |
| Middle frontal sulcus | 2.1036 | 2.3465 | 2.3892 | 1.822 | < .001 *** |  | 2.0728 | 2.3595 | 2.4050 | 17.380 | < .001 *** |
| Superior frontal sulcus | 2.1813 | 2.4735 | 2.5567 | 22.204 | < .001 *** |  | 2.2087 | 2.4885 | 2.5475 | 18.048 | < .001 *** |
| Sulcus intermedius primus (of Jensen) | 2.1183 | 2.4065 | 2.4358 | 19.997 | < .001 *** |  | 2.0598 | 2.3585 | 2.4383 | 22.805 | < .001 *** |
| Intraparietal sulcus (interparietal sulcus) and transverse parietal sulci | 1.9324 | 2.1690 | 2.2392 | 22.226 | < .001 *** |  | 1.8705 | 2.1460 | 2.1908 | 18.834 | < .001 *** |
| Middle occipital sulcus and lunatus sulcus | 1.8796 | 2.0445 | 2.1017 | 12.639 | < .001 *** |  | 1.9612 | 2.0805 | 2.1358 | 4.659 | .017 * |
| Superior occipital sulcus and transverse occipital sulcus | 1.8468 | 2.0750 | 2.1767 | 19.343 | < .001 *** |  | 1.8591 | 2.0320 | 2.1192 | 12.432 | < .001 *** |
| Anterior occipital sulcus and preoccipital notch (temporo-occipital incisure) | 2.0143 | 2.2455 | 2.3275 | 8.597 | .001 ** |  | 2.0460 | 2.2440 | 2.3608 | 6.253 | .005 ** |
| Lateral occipito-temporal sulcus | 2.2459 | 2.3985 | 2.3958 | 4.338 | .022 * |  | 2.2681 | 2.4020 | 2.3533 | 3.286 | .052 |
| Medial occipito-temporal sulcus (collateral sulcus) and lingual sulcus | 2.0413 | 2.2500 | 2.3733 | 12.214 | < .001 *** |  | 1.9871 | 2.2435 | 2.2808 | 22.110 | < .001 *** |
| Lateral orbital sulcus | 2.0541 | 2.3730 | 2.3742 | 13.353 | < .001 *** |  | 2.0881 | 2.3495 | 2.3692 | 11.571 | < .001 *** |
| Medial orbital sulcus (olfactory sulcus) | 2.2213 | 2.5100 | 2.6533 | 11.503 | < .001 *** |  | 2.2679 | 2.4925 | 2.5725 | 7.950 | .001 ** |
| Orbital sulci (H-shaped sulci) | 2.2502 | 2.4960 | 2.6167 | 23.928 | < .001 *** |  | 2.2880 | 2.5415 | 2.6117 | 22.165 | < .001 *** |
| Parieto-occipital sulcus (or ﬁssure) | 1.8556 | 2.1055 | 2.2150 | 17.495 | < .001 *** |  | 1.9111 | 2.0945 | 2.1633 | 9.813 | < .001 *** |
| Pericallosal sulcus (S of corpus callosum) | 1.9032 | 1.9300 | 1.9358 | .131 | .877 |  | 1.8170 | 1.9355 | 1.8467 | 2.153 | .137 |
| Postcentral sulcus | 1.7954 | 2.1360 | 2.1792 | 25.056 | < .001 *** |  | 1.7352 | 2.0915 | 2.1733 | 31.854 | < .001 *** |
| Inferior part of the precentral sulcus | 2.0758 | 2.4240 | 2.4992 | 18.692 | < .001 *** |  | 2.0500 | 2.4105 | 2.5017 | 19.019 | < .001 *** |
| Superior part of the precentral sulcus | 1.9461 | 2.2710 | 2.3708 | 13.839 | < .001 *** |  | 1.9511 | 2.2575 | 2.3800 | 16.291 | < .001 *** |
| Suborbital sulcus (sulcus rostrales. supraorbital sulcus) | 2.2444 | 2.4395 | 2.4483 | 4.279 | .023 * |  | 2.1933 | 2.4585 | 2.5033 | 5.811 | .007 ** |
| Subparietal sulcus | 2.0524 | 2.3370 | 2.3917 | 16.540 | < .001 *** |  | 2.1124 | 2.3290 | 2.3750 | 17.358 | < .001 *** |
| Inferior temporal sulcus | 2.2942 | 2.5145 | 2.4692 | 11.099 | < .001 *** |  | 2.3290 | 2.4825 | 2.5342 | 6.213 | .005 ** |
| Superior temporal sulcus (parallel sulcus) | 2.2425 | 2.4665 | 2.4942 | 13.401 | < .001 *** |  | 2.2074 | 2.5375 | 2.5517 | 26.185 | < .001 *** |
| Transverse temporal sulcus | 1.6573 | 2.1615 | 2.1567 | 15.369 | < .001 *** |  | 1.6590 | 2.1400 | 2.2100 | 15.677 | < .001 *** |

Supplementary Table 5: **Overview of all results of the cortical thickness ROI analysis after adjustment of data in order to minimize scanner effects based on the validational cohort.** Individual subject data was adjusted using the calculated scanner quotient before analysis and p-values of individual analyses were adjusted using Benjamini-Hochberg correction for multiple testing. Statistically significant results were marked with * for P < .05, ** for P < .01 and *** for P < .001.

| **Tract** | **Group** | **N** | **Normalized tract volume** | | | | |  |  | **Normalized tract length** | | | | |  |
| --- | --- | --- | --- | --- | --- | --- | --- | --- | --- | --- | --- | --- | --- | --- | --- |
|  |  |  | **Mean** | **Confidence Interval** | | **p value acute vs. Controls** | **p value acute vs. Recovered** | **p value recovered vs. Controls** |  | **Acutely ill  COVID-19 patients** | **Confidence Interval** | | **p value acute vs. Controls** | **p value acute vs. Recovered** | **p value recovered vs. Controls** |
| AC | Controls | 12 | .004 | .002 | .007 | 1.000 | 1.000 | 1.000 |  | 2.69171E-05 | 2.01553E-05 | 3.36788E-05 | .113 | .009 | 1.000 |
|  | Recovered | 18 | .005 | .003 | .008 |  |  |  |  | 2.96529E-05 | 2.30708E-05 | 3.6235E-05 |  |  |  |
|  | Acute | 9 | .003 | .001 | .005 |  |  |  |  | 1.54241E-05 | 1.17806E-05 | 1.90676E-05 |  |  |  |
| CBP L | Controls | 12 | .001 | .000 | .001 | .004 | .611 | .296 |  | 1.93178E-05 | 1.41036E-05 | 2.45319E-05 | 1.000 | 1.000 | 1.000 |
|  | Recovered | 20 | .001 | .001 | .001 |  |  |  |  | 1.77979E-05 | 1.7048E-05 | 1.85479E-05 |  |  |  |
|  | Acute | 13 | .001 | .001 | .002 |  |  |  |  | 1.73537E-05 | 1.57172E-05 | 1.89901E-05 |  |  |  |
| FX L | Controls | 11 | .003 | .002 | .004 | .208 | .109 | 1.000 |  | 4.43319E-05 | 3.95707E-05 | 4.9093E-05 | 1.000 | 1.000 | 1.000 |
|  | Recovered | 18 | .003 | .002 | .004 |  |  |  |  | 4.33938E-05 | 4.00391E-05 | 4.67485E-05 |  |  |  |
|  | Acute | 9 | .002 | .001 | .002 |  |  |  |  | 3.90912E-05 | 3.34626E-05 | 4.47197E-05 |  |  |  |
| OR L | Controls | 12 | .011 | .010 | .012 | .064 | .061 | 1.000 |  | 4.16611E-05 | 3.91111E-05 | 4.42111E-05 | .114 | 1.000 | 1.000 |
|  | Recovered | 20 | .011 | .011 | .012 |  |  |  |  | 3.99804E-05 | 3.87662E-05 | 4.11946E-05 |  |  |  |
|  | Acute | 14 | .010 | .009 | .011 |  |  |  |  | 3.86226E-05 | 3.69985E-05 | 4.02467E-05 |  |  |  |
| UF L | Controls | 12 | .009 | .009 | .010 | .266 | .243 | 1.000 |  | 3.36327E-05 | 3.14816E-05 | 3.57838E-05 | .700 | 1.000 | 1.000 |
|  | Recovered | 20 | .009 | .008 | .010 |  |  |  |  | 3.2198E-05 | 3.04586E-05 | 3.39374E-05 |  |  |  |
|  | Acute | 14 | .008 | .007 | .009 |  |  |  |  | 3.07559E-05 | 2.83651E-05 | 3.31468E-05 |  |  |  |
| CBT L | Controls | 12 | .003 | .002 | .003 | 1.000 | 1.000 | 1.000 |  | 1.50579E-05 | 1.36584E-05 | 1.64574E-05 | 1.000 | 1.000 | 1.000 |
|  | Recovered | 20 | .003 | .003 | .003 |  |  |  |  | 1.52165E-05 | 1.43175E-05 | 1.61155E-05 |  |  |  |
|  | Acute | 14 | .003 | .002 | .003 |  |  |  |  | 1.58048E-05 | 1.39269E-05 | 1.76827E-05 |  |  |  |
| IFO L | Controls | 12 | .020 | .017 | .022 | .994 | 1.000 | 1.000 |  | 4.70405E-05 | 4.35045E-05 | 5.05766E-05 | 1.000 | 1.000 | 1.000 |
|  | Recovered | 20 | .019 | .018 | .020 |  |  |  |  | 4.62113E-05 | 4.40112E-05 | 4.84114E-05 |  |  |  |
|  | Acute | 14 | .018 | .017 | .019 |  |  |  |  | 4.44432E-05 | 4.27274E-05 | 4.61589E-05 |  |  |  |
| SLF1 L | Controls | 8 | .002 | -.001 | .005 | 1.000 | 1.000 | 1.000 |  | 2.41906E-05 | 1.98035E-05 | 2.85776E-05 | .244 | .032 | 1.000 |
|  | Recovered | 18 | .003 | .001 | .004 |  |  |  |  | 2.45401E-05 | 2.19513E-05 | 2.71288E-05 |  |  |  |
|  | Acute | 13 | .002 | .001 | .002 |  |  |  |  | 1.96822E-05 | 1.78905E-05 | 2.14739E-05 |  |  |  |
| VOF L | Controls | 12 | .006 | .006 | .007 | .859 | .170 | 1.000 |  | 1.37739E-05 | 1.28314E-05 | 1.47165E-05 | 1.000 | 1.000 | 1.000 |
|  | Recovered | 20 | .006 | .006 | .007 |  |  |  |  | 1.37213E-05 | 1.30402E-05 | 1.44024E-05 |  |  |  |
|  | Acute | 14 | .006 | .005 | .006 |  |  |  |  | 1.31942E-05 | 1.21004E-05 | 1.4288E-05 |  |  |  |
| CST L | Controls | 12 | .010 | .009 | .011 | .020 | .021 | 1.000 |  | 5.47947E-05 | 5.22547E-05 | 5.73346E-05 | 1.000 | 1.000 | 1.000 |
|  | Recovered | 20 | .010 | .010 | .010 |  |  |  |  | 5.48038E-05 | 5.27874E-05 | 5.68202E-05 |  |  |  |
|  | Acute | 14 | .008 | .007 | .009 |  |  |  |  | 5.29234E-05 | 5.06795E-05 | 5.51673E-05 |  |  |  |
| ILF L | Controls | 12 | .010 | .009 | .011 | 1.000 | 1.000 | 1.000 |  | 3.36655E-05 | 2.94966E-05 | 3.78344E-05 | 1.000 | .137 | 1.000 |
|  | Recovered | 20 | .009 | .008 | .010 |  |  |  |  | 3.15106E-05 | 2.90659E-05 | 3.39553E-05 |  |  |  |
|  | Acute | 14 | .009 | .009 | .010 |  |  |  |  | 3.59954E-05 | 3.34046E-05 | 3.85862E-05 |  |  |  |
| SLF2 L | Controls | 12 | .006 | .003 | .008 | 1.000 | 1.000 | 1.000 |  | 2.9377E-05 | 2.66295E-05 | 3.21244E-05 | .037 | .153 | 1.000 |
|  | Recovered | 18 | .005 | .003 | .006 |  |  |  |  | 2.81053E-05 | 2.61998E-05 | 3.00108E-05 |  |  |  |
|  | Acute | 13 | .004 | .002 | .005 |  |  |  |  | 2.42315E-05 | 2.17477E-05 | 2.67153E-05 |  |  |  |
| FAs L | Controls | 12 | .007 | .007 | .008 | .253 | .024 | 1.000 |  | 2.25173E-05 | 2.07636E-05 | 2.42709E-05 | 1.000 | 1.000 | 1.000 |
|  | Recovered | 20 | .007 | .007 | .008 |  |  |  |  | 2.26188E-05 | 2.15529E-05 | 2.36847E-05 |  |  |  |
|  | Acute | 14 | .006 | .006 | .007 |  |  |  |  | 2.18708E-05 | 2.01634E-05 | 2.35783E-05 |  |  |  |
| MCP | Controls | 12 | .011 | .009 | .012 | .744 | .050 | 1.000 |  | 3.20587E-05 | 2.81988E-05 | 3.59185E-05 | 1.000 | 1.000 | 1.000 |
|  | Recovered | 20 | .011 | .010 | .013 |  |  |  |  | 3.33869E-05 | 3.10695E-05 | 3.57042E-05 |  |  |  |
|  | Acute | 14 | .009 | .008 | .010 |  |  |  |  | 3.07145E-05 | 2.83589E-05 | 3.307E-05 |  |  |  |
| SLF3 L | Controls | 12 | .010 | .009 | .010 | .716 | .019 | 1.000 |  | 2.74391E-05 | 2.57628E-05 | 2.91154E-05 | .119 | .005 | 1.000 |
|  | Recovered | 20 | .010 | .009 | .011 |  |  |  |  | 2.84158E-05 | 2.68621E-05 | 2.99694E-05 |  |  |  |
|  | Acute | 14 | .008 | .008 | .009 |  |  |  |  | 2.36649E-05 | 2.14508E-05 | 2.58789E-05 |  |  |  |
| FMA | Controls | 12 | .015 | .014 | .016 | .125 | .015 | 1.000 |  | 5.26589E-05 | 4.96898E-05 | 5.5628E-05 | 1.000 | 1.000 | 1.000 |
|  | Recovered | 20 | .015 | .014 | .016 |  |  |  |  | 5.39431E-05 | 5.17245E-05 | 5.61616E-05 |  |  |  |
|  | Acute | 14 | .013 | .012 | .014 |  |  |  |  | 5.24364E-05 | 5.00059E-05 | 5.48669E-05 |  |  |  |
| STR L | Controls | 12 | .010 | .010 | .011 | 1.000 | .068 | 1.000 |  | 2.74968E-05 | 2.61057E-05 | 2.88879E-05 | 1.000 | 1.000 | 1.000 |
|  | Recovered | 20 | .011 | .010 | .011 |  |  |  |  | 2.73953E-05 | 2.63639E-05 | 2.84268E-05 |  |  |  |
|  | Acute | 14 | .009 | .009 | .010 |  |  |  |  | 2.65226E-05 | 2.52498E-05 | 2.77955E-05 |  |  |  |
| MdLF L | Controls | 12 | .013 | .012 | .014 | 1.000 | .363 | 1.000 |  | 4.28739E-05 | 4.05585E-05 | 4.51894E-05 | 1.000 | 1.000 | 1.000 |
|  | Recovered | 20 | .013 | .012 | .014 |  |  |  |  | 4.25598E-05 | 4.11443E-05 | 4.39752E-05 |  |  |  |
|  | Acute | 14 | .012 | .011 | .012 |  |  |  |  | 4.07524E-05 | 3.88349E-05 | 4.26699E-05 |  |  |  |
| AF L | Controls | 12 | .016 | .014 | .017 | .077 | .023 | 1.000 |  | 2.84355E-05 | 2.65895E-05 | 3.02815E-05 | .121 | .223 | 1.000 |
|  | Recovered | 20 | .016 | .015 | .017 |  |  |  |  | 2.79064E-05 | 2.6716E-05 | 2.90968E-05 |  |  |  |
|  | Acute | 14 | .013 | .012 | .014 |  |  |  |  | 2.57461E-05 | 2.43117E-05 | 2.71804E-05 |  |  |  |
| AR L | Controls | 10 | .001 | .001 | .002 | 1.000 | 1.000 | 1.000 |  | 2.70975E-05 | 2.47922E-05 | 2.94029E-05 | 1.000 | .388 | 1.000 |
|  | Recovered | 17 | .001 | .001 | .002 |  |  |  |  | 2.824E-05 | 2.63068E-05 | 3.01732E-05 |  |  |  |
|  | Acute | 14 | .002 | .001 | .002 |  |  |  |  | 2.54058E-05 | 2.31715E-05 | 2.76401E-05 |  |  |  |
| ATR L | Controls | 12 | .011 | .010 | .013 | .010 | .010 | 1.000 |  | 3.13635E-05 | 2.91273E-05 | 3.35998E-05 | 1.000 | 1.000 | 1.000 |
|  | Recovered | 20 | .011 | .010 | .012 |  |  |  |  | 3.13889E-05 | 3.00522E-05 | 3.27255E-05 |  |  |  |
|  | Acute | 14 | .009 | .008 | .010 |  |  |  |  | 3.0755E-05 | 2.92619E-05 | 3.22481E-05 |  |  |  |
| CBD L | Controls | 12 | .006 | .005 | .007 | .292 | 1.000 | 1.000 |  | 4.37772E-05 | 4.00494E-05 | 4.7505E-05 | .050 | 1.000 | .867 |
|  | Recovered | 20 | .006 | .005 | .006 |  |  |  |  | 4.02525E-05 | 3.8136E-05 | 4.2369E-05 |  |  |  |
|  | Acute | 14 | .005 | .004 | .006 |  |  |  |  | 3.74343E-05 | 3.42045E-05 | 4.06641E-05 |  |  |  |
| CBP R | Controls | 11 | .001 | .001 | .001 | 1.000 | 1.000 | 1.000 |  | 1.67837E-05 | 1.55889E-05 | 1.79785E-05 | 1.000 | 1.000 | 1.000 |
|  | Recovered | 20 | .001 | .001 | .001 |  |  |  |  | 1.76734E-05 | 1.6762E-05 | 1.85849E-05 |  |  |  |
|  | Acute | 14 | .001 | .001 | .001 |  |  |  |  | 1.66134E-05 | 1.51677E-05 | 1.80591E-05 |  |  |  |
| FX R | Controls | 10 | .003 | .002 | .004 | 1.000 | 1.000 | 1.000 |  | 4.14686E-05 | 3.72149E-05 | 4.57223E-05 | .104 | .489 | 1.000 |
|  | Recovered | 19 | .002 | .001 | .003 |  |  |  |  | 3.91032E-05 | 3.6104E-05 | 4.21024E-05 |  |  |  |
|  | Acute | 11 | .002 | .001 | .003 |  |  |  |  | 3.45326E-05 | 3.07378E-05 | 3.83273E-05 |  |  |  |
| OR R | Controls | 12 | .013 | .012 | .013 | <.001 | <.001 | .696 |  | 4.15252E-05 | 3.86802E-05 | 4.43703E-05 | .989 | 1.000 | 1.000 |
|  | Recovered | 20 | .012 | .011 | .012 |  |  |  |  | 4.0652E-05 | 3.9292E-05 | 4.20119E-05 |  |  |  |
|  | Acute | 14 | .010 | .009 | .010 |  |  |  |  | 3.9512E-05 | 3.79994E-05 | 4.10245E-05 |  |  |  |
| UF R | Controls | 12 | .008 | .007 | .009 | 1.000 | .326 | 1.000 |  | 2.97153E-05 | 2.77048E-05 | 3.17258E-05 | 1.000 | 1.000 | 1.000 |
|  | Recovered | 20 | .009 | .008 | .009 |  |  |  |  | 2.87284E-05 | 2.72005E-05 | 3.02564E-05 |  |  |  |
|  | Acute | 14 | .008 | .007 | .008 |  |  |  |  | 2.81734E-05 | 2.55109E-05 | 3.08359E-05 |  |  |  |
| CBT R | Controls | 12 | .003 | .002 | .003 | 1.000 | 1.000 | 1.000 |  | 1.55677E-05 | 1.43538E-05 | 1.67817E-05 | 1.000 | 1.000 | 1.000 |
|  | Recovered | 20 | .003 | .002 | .003 |  |  |  |  | 1.55652E-05 | 1.44971E-05 | 1.66334E-05 |  |  |  |
|  | Acute | 14 | .003 | .002 | .003 |  |  |  |  | 1.59834E-05 | 1.47202E-05 | 1.72466E-05 |  |  |  |
| IFO R | Controls | 12 | .020 | .017 | .022 | .001 | .007 | 1.000 |  | 4.39227E-05 | 3.99905E-05 | 4.78548E-05 | 1.000 | 1.000 | 1.000 |
|  | Recovered | 20 | .019 | .018 | .020 |  |  |  |  | 4.43481E-05 | 4.2308E-05 | 4.63881E-05 |  |  |  |
|  | Acute | 14 | .016 | .015 | .017 |  |  |  |  | 4.51194E-05 | 4.32991E-05 | 4.69396E-05 |  |  |  |
| SLF1 R | Controls | 8 | .002 | .000 | .005 | 1.000 | 1.000 | 1.000 |  | 2.58228E-05 | 2.18352E-05 | 2.98103E-05 | 1.000 | 1.000 | 1.000 |
|  | Recovered | 17 | .003 | .002 | .005 |  |  |  |  | 2.57579E-05 | 2.31652E-05 | 2.83507E-05 |  |  |  |
|  | Acute | 14 | .002 | .001 | .002 |  |  |  |  | 2.34502E-05 | 2.11852E-05 | 2.57153E-05 |  |  |  |
| VOF R | Controls | 12 | .006 | .006 | .007 | .066 | .102 | 1.000 |  | 1.44002E-05 | 1.3233E-05 | 1.55675E-05 | 1.000 | 1.000 | 1.000 |
|  | Recovered | 20 | .006 | .005 | .007 |  |  |  |  | 1.43202E-05 | 1.34169E-05 | 1.52236E-05 |  |  |  |
|  | Acute | 14 | .005 | .004 | .005 |  |  |  |  | 1.32765E-05 | 1.23227E-05 | 1.42303E-05 |  |  |  |
| CST R | Controls | 12 | .010 | .010 | .011 | .004 | .001 | 1.000 |  | 5.37836E-05 | 5.10056E-05 | 5.65615E-05 | 1.000 | 1.000 | 1.000 |
|  | Recovered | 20 | .010 | .010 | .011 |  |  |  |  | 5.34251E-05 | 5.14549E-05 | 5.53953E-05 |  |  |  |
|  | Acute | 14 | .008 | .008 | .009 |  |  |  |  | 5.18342E-05 | 4.98258E-05 | 5.38427E-05 |  |  |  |
| ILF R | Controls | 12 | .010 | .009 | .011 | 1.000 | 1.000 | 1.000 |  | 3.07532E-05 | 2.82085E-05 | 3.32979E-05 | 1.000 | 1.000 | 1.000 |
|  | Recovered | 20 | .010 | .009 | .010 |  |  |  |  | 3.08092E-05 | 2.89425E-05 | 3.26759E-05 |  |  |  |
|  | Acute | 14 | .009 | .008 | .010 |  |  |  |  | 3.24781E-05 | 2.9577E-05 | 3.53792E-05 |  |  |  |
| SLF2 R | Controls | 12 | .005 | .002 | .008 | 1.000 | .901 | .191 |  | 2.89783E-05 | 2.56927E-05 | 3.22639E-05 | 1.000 | 1.000 | 1.000 |
|  | Recovered | 20 | .007 | .006 | .009 |  |  |  |  | 3.0534E-05 | 2.84464E-05 | 3.26215E-05 |  |  |  |
|  | Acute | 14 | .006 | .004 | .007 |  |  |  |  | 2.8237E-05 | 2.60073E-05 | 3.04668E-05 |  |  |  |
| FAs R | Controls | 12 | .006 | .005 | .007 | 1.000 | .007 | .348 |  | 2.14084E-05 | 1.93551E-05 | 2.34618E-05 | 1.000 | 1.000 | 1.000 |
|  | Recovered | 20 | .007 | .007 | .008 |  |  |  |  | 2.19234E-05 | 2.12033E-05 | 2.26435E-05 |  |  |  |
|  | Acute | 14 | .006 | .006 | .006 |  |  |  |  | 2.12427E-05 | 2.00868E-05 | 2.23986E-05 |  |  |  |
| SLF3 R | Controls | 12 | .010 | .007 | .012 | .232 | .001 | 1.000 |  | 2.77969E-05 | 2.58191E-05 | 2.97748E-05 | 1.000 | 1.000 | 1.000 |
|  | Recovered | 20 | .011 | .010 | .012 |  |  |  |  | 2.82004E-05 | 2.65624E-05 | 2.98385E-05 |  |  |  |
|  | Acute | 14 | .008 | .007 | .008 |  |  |  |  | 2.69802E-05 | 2.56425E-05 | 2.83178E-05 |  |  |  |
| FMI | Controls | 12 | .017 | .016 | .019 | .003 | .002 | 1.000 |  | 3.18093E-05 | 3.04869E-05 | 3.31317E-05 | 1.000 | 1.000 | 1.000 |
|  | Recovered | 20 | .017 | .016 | .018 |  |  |  |  | 3.27158E-05 | 3.15622E-05 | 3.38695E-05 |  |  |  |
|  | Acute | 14 | .014 | .013 | .015 |  |  |  |  | 3.29051E-05 | 3.13207E-05 | 3.44896E-05 |  |  |  |
| STR R | Controls | 12 | .009 | .008 | .010 | 1.000 | .045 | .040 |  | 2.8547E-05 | 2.68591E-05 | 3.02349E-05 | 1.000 | 1.000 | 1.000 |
|  | Recovered | 20 | .010 | .010 | .011 |  |  |  |  | 2.86347E-05 | 2.76005E-05 | 2.96688E-05 |  |  |  |
|  | Acute | 14 | .009 | .008 | .010 |  |  |  |  | 2.73795E-05 | 2.59127E-05 | 2.88464E-05 |  |  |  |
| MdLF R | Controls | 12 | .013 | .012 | .014 | .482 | .235 | 1.000 |  | 4.15907E-05 | 3.89566E-05 | 4.42247E-05 | .276 | 1.000 | 1.000 |
|  | Recovered | 20 | .013 | .012 | .014 |  |  |  |  | 4.02529E-05 | 3.86526E-05 | 4.18532E-05 |  |  |  |
|  | Acute | 14 | .012 | .011 | .012 |  |  |  |  | 3.87882E-05 | 3.72536E-05 | 4.03227E-05 |  |  |  |
| AF R | Controls | 12 | .016 | .014 | .019 | .058 | .028 | 1.000 |  | 2.79078E-05 | 2.5481E-05 | 3.03346E-05 | .030 | .060 | 1.000 |
|  | Recovered | 20 | .016 | .015 | .017 |  |  |  |  | 2.72221E-05 | 2.55668E-05 | 2.88774E-05 |  |  |  |
|  | Acute | 14 | .014 | .013 | .015 |  |  |  |  | 2.41479E-05 | 2.27517E-05 | 2.5544E-05 |  |  |  |
| AR R | Controls | 12 | .002 | .001 | .002 | 1.000 | 1.000 | 1.000 |  | 2.34132E-05 | 2.12705E-05 | 2.5556E-05 | 1.000 | 1.000 | .681 |
|  | Recovered | 20 | .002 | .002 | .003 |  |  |  |  | 2.55391E-05 | 2.40731E-05 | 2.70051E-05 |  |  |  |
|  | Acute | 13 | .002 | .001 | .003 |  |  |  |  | 2.46372E-05 | 2.27101E-05 | 2.65643E-05 |  |  |  |
| ATR R | Controls | 12 | .012 | .011 | .013 | .003 | .008 | 1.000 |  | 3.07987E-05 | 2.8715E-05 | 3.28824E-05 | 1.000 | 1.000 | 1.000 |
|  | Recovered | 20 | .011 | .011 | .012 |  |  |  |  | 3.05458E-05 | 2.92603E-05 | 3.18312E-05 |  |  |  |
|  | Acute | 14 | .010 | .009 | .010 |  |  |  |  | 3.17469E-05 | 3.05088E-05 | 3.2985E-05 |  |  |  |
| CBD R | Controls | 12 | .005 | .005 | .006 | 1.000 | 1.000 | 1.000 |  | 4.11142E-05 | 3.76989E-05 | 4.45296E-05 | 1.000 | 1.000 | 1.000 |
|  | Recovered | 20 | .006 | .005 | .006 |  |  |  |  | 4.04409E-05 | 3.81297E-05 | 4.2752E-05 |  |  |  |
|  | Acute | 14 | .005 | .003 | .006 |  |  |  |  | 4.02119E-05 | 3.57397E-05 | 4.4684E-05 |  |  |  |

Supplementary Table 6: **Overview of the results of probabilistic tractography (normalized tract volume and normalized tract length) after adjustment of data in order to minimize scanner effects based on the validational cohort.** Mean values are shown for each tract for the group of controls, recovered and acute cases as well as 95% confidence intervals. P-values of post-hoc t-tests adjusted using Bonferroni correction for multiple testing are shown for comparisons between acute cases and controls, acute and recovered cases as well as recovered cases and controls. Abbreviations: B = bilateral, L = left, R = right. AC = Anterior Commissure; AF= Arcuate Fasciculus; AR= Acoustic Radiation; ATR = Anterior Thalamic Radiation; CBD = dorsal Cingulum subsection; CBP = peri-genual Cingulum subsection; CBT = temporal Cingulum subsection; CST = Corticospinal Tract; FAs = Frontal Aslant; FMA = Forceps Major; FMI = Forceps Minor; FX = Fornix; ILF = Inferior Longitudinal Fasciculus; IFO = Inferior Fronto-Occipital Fasciculus; MCP = Middle Cerebellar Peduncle; MdLF = Middle Longitudinal Fasciculus; OR = Optic Radiation; SLF1/ 2/ 3 = Superior Longitudinal Fasciculus 1/ 2/ 3; STR = Superior Thalamic Radiation; UF = Uncinate Fasciculus; VOF = Vertical Occipital Fasciculus.

| **Tract** | **Group** | **N** | **Fractional Anisotropy** | | | | |  |  | **Mean Diffusivity** | | | | |  |
| --- | --- | --- | --- | --- | --- | --- | --- | --- | --- | --- | --- | --- | --- | --- | --- |
|  |  |  | **Mean** | **Confidence Interval** | | **p value acute vs. Controls** | **p value acute vs. Recovered** | **p value recovered vs. Controls** |  | **Acutely ill  COVID-19 patients** | **Confidence Interval** | | **p value acute vs. Controls** | **p value acute vs. Recovered** | **p value recovered vs. Controls** |
| AC | Controls | 12 | .372 | .345 | .399 | .576 | .003 | .627 |  | .00098 | .00092 | .00104 | <.001 | <.001 | 1.000 |
|  | Recovered | 18 | .398 | .377 | .419 |  |  |  |  | .00102 | .00095 | .00109 |  |  |  |
|  | Acute | 9 | .341 | .319 | .362 |  |  |  |  | .00124 | .00114 | .00134 |  |  |  |
| CBP L | Controls | 12 | .396 | .375 | .416 | .083 | .040 | 1.000 |  | .00080 | .00079 | .00082 | .745 | .035 | 1.000 |
|  | Recovered | 20 | .395 | .379 | .411 |  |  |  |  | .00079 | .00078 | .00080 |  |  |  |
|  | Acute | 13 | .363 | .347 | .379 |  |  |  |  | .00083 | .00080 | .00086 |  |  |  |
| FX L | Controls | 11 | .316 | .295 | .338 | 1.000 | .782 | 1.000 |  | .00129 | .00120 | .00137 | .004 | .003 | 1.000 |
|  | Recovered | 18 | .320 | .305 | .335 |  |  |  |  | .00131 | .00122 | .00140 |  |  |  |
|  | Acute | 9 | .297 | .272 | .322 |  |  |  |  | .00158 | .00144 | .00172 |  |  |  |
| OR L | Controls | 12 | .439 | .430 | .448 | .070 | .044 | 1.000 |  | .00085 | .00082 | .00087 | .133 | .011 | 1.000 |
|  | Recovered | 20 | .437 | .426 | .448 |  |  |  |  | .00083 | .00082 | .00085 |  |  |  |
|  | Acute | 14 | .408 | .389 | .427 |  |  |  |  | .00092 | .00087 | .00098 |  |  |  |
| UF L | Controls | 12 | .349 | .336 | .363 | 1.000 | .149 | 1.000 |  | .00087 | .00084 | .00089 | .012 | <.001 | 1.000 |
|  | Recovered | 20 | .359 | .352 | .367 |  |  |  |  | .00084 | .00083 | .00085 |  |  |  |
|  | Acute | 14 | .341 | .327 | .355 |  |  |  |  | .00095 | .00090 | .00099 |  |  |  |
| CBT L | Controls | 12 | .294 | .282 | .307 | 1.000 | .056 | 1.000 |  | .00092 | .00089 | .00095 | <.001 | <.001 | .658 |
|  | Recovered | 20 | .305 | .294 | .316 |  |  |  |  | .00089 | .00087 | .00090 |  |  |  |
|  | Acute | 14 | .284 | .271 | .296 |  |  |  |  | .00100 | .00097 | .00104 |  |  |  |
| IFO L | Controls | 12 | .418 | .409 | .427 | .048 | <.001 | 1.000 |  | .00083 | .00082 | .00084 | .012 | <.001 | 1.000 |
|  | Recovered | 20 | .425 | .413 | .437 |  |  |  |  | .00081 | .00080 | .00082 |  |  |  |
|  | Acute | 14 | .391 | .377 | .405 |  |  |  |  | .00090 | .00086 | .00094 |  |  |  |
| SLF1 L | Controls | 8 | .422 | .394 | .449 | 1.000 | 1.000 | 1.000 |  | .00080 | .00074 | .00087 | 1.000 | 1.000 | 1.000 |
|  | Recovered | 18 | .409 | .396 | .422 |  |  |  |  | .00081 | .00078 | .00084 |  |  |  |
|  | Acute | 13 | .393 | .362 | .424 |  |  |  |  | .00083 | .00075 | .00091 |  |  |  |
| VOF L | Controls | 12 | .296 | .282 | .309 | .656 | .005 | 1.000 |  | .00089 | .00086 | .00091 | 1.000 | .016 | 1.000 |
|  | Recovered | 20 | .305 | .296 | .315 |  |  |  |  | .00085 | .00085 | .00086 |  |  |  |
|  | Acute | 14 | .282 | .272 | .292 |  |  |  |  | .00092 | .00088 | .00097 |  |  |  |
| CST L | Controls | 12 | .437 | .423 | .450 | 1.000 | 1.000 | 1.000 |  | .00085 | .00083 | .00087 | 1.000 | .817 | 1.000 |
|  | Recovered | 20 | .436 | .428 | .445 |  |  |  |  | .00085 | .00083 | .00086 |  |  |  |
|  | Acute | 14 | .429 | .411 | .447 |  |  |  |  | .00089 | .00084 | .00094 |  |  |  |
| ILF L | Controls | 12 | .398 | .386 | .411 | .257 | .357 | 1.000 |  | .00081 | .00080 | .00083 | .115 | .004 | 1.000 |
|  | Recovered | 20 | .394 | .383 | .406 |  |  |  |  | .00080 | .00079 | .00081 |  |  |  |
|  | Acute | 14 | .375 | .359 | .391 |  |  |  |  | .00086 | .00083 | .00090 |  |  |  |
| SLF2 L | Controls | 12 | .353 | .335 | .371 | 1.000 | .203 | .706 |  | .00090 | .00086 | .00094 | 1.000 | <.001 | .023 |
|  | Recovered | 18 | .372 | .361 | .382 |  |  |  |  | .00083 | .00082 | .00085 |  |  |  |
|  | Acute | 13 | .348 | .331 | .366 |  |  |  |  | .00092 | .00089 | .00095 |  |  |  |
| FAs L | Controls | 12 | .334 | .324 | .344 | .115 | .001 | 1.000 |  | .00094 | .00091 | .00096 | .276 | <.001 | 1.000 |
|  | Recovered | 20 | .344 | .336 | .352 |  |  |  |  | .00090 | .00088 | .00092 |  |  |  |
|  | Acute | 14 | .309 | .292 | .326 |  |  |  |  | .00099 | .00095 | .00103 |  |  |  |
| MCP | Controls | 12 | .448 | .420 | .477 | 1.000 | 1.000 | 1.000 |  | .00085 | .00081 | .00088 | .066 | <.001 | .508 |
|  | Recovered | 20 | .450 | .426 | .475 |  |  |  |  | .00081 | .00078 | .00083 |  |  |  |
|  | Acute | 14 | .443 | .423 | .462 |  |  |  |  | .00091 | .00087 | .00095 |  |  |  |
| SLF3 L | Controls | 12 | .354 | .344 | .363 | .066 | .022 | 1.000 |  | .00090 | .00088 | .00092 | .077 | <.001 | 1.000 |
|  | Recovered | 20 | .354 | .346 | .362 |  |  |  |  | .00087 | .00085 | .00089 |  |  |  |
|  | Acute | 14 | .328 | .312 | .344 |  |  |  |  | .00098 | .00093 | .00103 |  |  |  |
| FMA | Controls | 12 | .440 | .416 | .464 | .164 | .462 | 1.000 |  | .00092 | .00089 | .00095 | .812 | .355 | 1.000 |
|  | Recovered | 20 | .432 | .419 | .444 |  |  |  |  | .00092 | .00090 | .00094 |  |  |  |
|  | Acute | 14 | .410 | .392 | .428 |  |  |  |  | .00097 | .00093 | .00102 |  |  |  |
| STR L | Controls | 12 | .400 | .388 | .411 | 1.000 | 1.000 | 1.000 |  | .00085 | .00084 | .00087 | 1.000 | 1.000 | 1.000 |
|  | Recovered | 20 | .400 | .394 | .407 |  |  |  |  | .00084 | .00082 | .00085 |  |  |  |
|  | Acute | 14 | .389 | .373 | .406 |  |  |  |  | .00088 | .00082 | .00094 |  |  |  |
| MdLF L | Controls | 12 | .400 | .388 | .413 | .004 | <.001 | 1.000 |  | .00085 | .00083 | .00086 | .041 | <.001 | 1.000 |
|  | Recovered | 20 | .401 | .392 | .409 |  |  |  |  | .00083 | .00082 | .00084 |  |  |  |
|  | Acute | 14 | .368 | .354 | .382 |  |  |  |  | .00091 | .00087 | .00095 |  |  |  |
| AF L | Controls | 12 | .386 | .378 | .393 | .066 | .004 | 1.000 |  | .00081 | .00080 | .00083 | .021 | <.001 | 1.000 |
|  | Recovered | 20 | .391 | .383 | .399 |  |  |  |  | .00079 | .00078 | .00080 |  |  |  |
|  | Acute | 14 | .360 | .343 | .376 |  |  |  |  | .00087 | .00084 | .00090 |  |  |  |
| AR L | Controls | 10 | .431 | .402 | .460 | 1.000 | 1.000 | 1.000 |  | .00080 | .00076 | .00084 | .107 | .106 | 1.000 |
|  | Recovered | 17 | .433 | .410 | .456 |  |  |  |  | .00081 | .00079 | .00083 |  |  |  |
|  | Acute | 14 | .407 | .381 | .434 |  |  |  |  | .00088 | .00083 | .00093 |  |  |  |
| ATR L | Controls | 12 | .392 | .383 | .401 | 1.000 | 1.000 | 1.000 |  | .00080 | .00078 | .00082 | .017 | <.001 | 1.000 |
|  | Recovered | 20 | .394 | .387 | .401 |  |  |  |  | .00079 | .00078 | .00080 |  |  |  |
|  | Acute | 14 | .387 | .375 | .398 |  |  |  |  | .00087 | .00083 | .00091 |  |  |  |
| CBD L | Controls | 12 | .401 | .389 | .414 | .535 | .016 | 1.000 |  | .00078 | .00077 | .00080 | .297 | .048 | 1.000 |
|  | Recovered | 20 | .412 | .400 | .424 |  |  |  |  | .00077 | .00076 | .00079 |  |  |  |
|  | Acute | 14 | .379 | .360 | .398 |  |  |  |  | .00084 | .00079 | .00089 |  |  |  |
| CBP R | Controls | 11 | .357 | .338 | .376 | .996 | .189 | 1.000 |  | .00080 | .00079 | .00082 | .879 | .010 | 1.000 |
|  | Recovered | 20 | .364 | .346 | .382 |  |  |  |  | .00078 | .00078 | .00079 |  |  |  |
|  | Acute | 14 | .331 | .307 | .355 |  |  |  |  | .00083 | .00080 | .00086 |  |  |  |
| FX R | Controls | 10 | .318 | .301 | .335 | 1.000 | .183 | 1.000 |  | .00124 | .00117 | .00130 | <.001 | <.001 | 1.000 |
|  | Recovered | 19 | .326 | .306 | .347 |  |  |  |  | .00129 | .00123 | .00136 |  |  |  |
|  | Acute | 11 | .296 | .277 | .316 |  |  |  |  | .00155 | .00146 | .00165 |  |  |  |
| OR R | Controls | 12 | .436 | .424 | .447 | .005 | <.001 | 1.000 |  | .00086 | .00083 | .00088 | .028 | <.001 | 1.000 |
|  | Recovered | 20 | .441 | .432 | .450 |  |  |  |  | .00084 | .00083 | .00085 |  |  |  |
|  | Acute | 14 | .404 | .390 | .418 |  |  |  |  | .00091 | .00088 | .00094 |  |  |  |
| UF R | Controls | 12 | .345 | .335 | .356 | 1.000 | .237 | 1.000 |  | .00087 | .00084 | .00089 | .109 | <.001 | 1.000 |
|  | Recovered | 20 | .359 | .349 | .369 |  |  |  |  | .00084 | .00083 | .00086 |  |  |  |
|  | Acute | 14 | .340 | .324 | .356 |  |  |  |  | .00092 | .00089 | .00096 |  |  |  |
| CBT R | Controls | 12 | .311 | .296 | .325 | .007 | <.001 | 1.000 |  | .00093 | .00091 | .00095 | .001 | <.001 | 1.000 |
|  | Recovered | 20 | .315 | .306 | .324 |  |  |  |  | .00092 | .00090 | .00093 |  |  |  |
|  | Acute | 14 | .280 | .266 | .293 |  |  |  |  | .00103 | .00099 | .00108 |  |  |  |
| IFO R | Controls | 12 | .421 | .411 | .432 | .010 | <.001 | 1.000 |  | .00083 | .00082 | .00084 | .014 | <.001 | 1.000 |
|  | Recovered | 20 | .424 | .415 | .434 |  |  |  |  | .00082 | .00081 | .00083 |  |  |  |
|  | Acute | 14 | .386 | .369 | .404 |  |  |  |  | .00090 | .00086 | .00094 |  |  |  |
| SLF1 R | Controls | 8 | .400 | .362 | .438 | 1.000 | 1.000 | 1.000 |  | .00085 | .00078 | .00092 | 1.000 | 1.000 | 1.000 |
|  | Recovered | 17 | .382 | .362 | .403 |  |  |  |  | .00083 | .00078 | .00088 |  |  |  |
|  | Acute | 14 | .397 | .370 | .423 |  |  |  |  | .00083 | .00076 | .00090 |  |  |  |
| VOF R | Controls | 12 | .307 | .296 | .317 | .239 | .149 | 1.000 |  | .00090 | .00087 | .00093 | .364 | .004 | 1.000 |
|  | Recovered | 20 | .306 | .297 | .315 |  |  |  |  | .00087 | .00086 | .00089 |  |  |  |
|  | Acute | 14 | .286 | .270 | .301 |  |  |  |  | .00095 | .00091 | .00100 |  |  |  |
| CST R | Controls | 12 | .442 | .432 | .453 | 1.000 | 1.000 | 1.000 |  | .00084 | .00082 | .00087 | 1.000 | 1.000 | 1.000 |
|  | Recovered | 20 | .441 | .432 | .449 |  |  |  |  | .00084 | .00082 | .00085 |  |  |  |
|  | Acute | 14 | .442 | .420 | .465 |  |  |  |  | .00086 | .00079 | .00092 |  |  |  |
| ILF R | Controls | 12 | .401 | .383 | .419 | 1.000 | .238 | 1.000 |  | .00080 | .00079 | .00082 | .119 | .011 | 1.000 |
|  | Recovered | 20 | .407 | .392 | .422 |  |  |  |  | .00080 | .00079 | .00081 |  |  |  |
|  | Acute | 14 | .382 | .364 | .400 |  |  |  |  | .00085 | .00082 | .00087 |  |  |  |
| SLF2 R | Controls | 12 | .386 | .366 | .405 | .009 | .223 | 1.000 |  | .00084 | .00080 | .00088 | .154 | .230 | 1.000 |
|  | Recovered | 20 | .368 | .355 | .382 |  |  |  |  | .00085 | .00083 | .00088 |  |  |  |
|  | Acute | 14 | .343 | .324 | .362 |  |  |  |  | .00092 | .00087 | .00098 |  |  |  |
| FAs R | Controls | 12 | .339 | .325 | .352 | .468 | .045 | 1.000 |  | .00091 | .00088 | .00095 | .539 | .046 | 1.000 |
|  | Recovered | 20 | .344 | .335 | .354 |  |  |  |  | .00090 | .00087 | .00093 |  |  |  |
|  | Acute | 14 | .319 | .303 | .334 |  |  |  |  | .00096 | .00093 | .00099 |  |  |  |
| SLF3 R | Controls | 12 | .356 | .348 | .363 | 1.000 | .164 | 1.000 |  | .00092 | .00090 | .00094 | 1.000 | .056 | .211 |
|  | Recovered | 20 | .363 | .355 | .370 |  |  |  |  | .00086 | .00085 | .00088 |  |  |  |
|  | Acute | 14 | .345 | .331 | .359 |  |  |  |  | .00092 | .00088 | .00097 |  |  |  |
| FMI | Controls | 12 | .419 | .402 | .436 | .585 | .004 | 1.000 |  | .00086 | .00084 | .00089 | .131 | .001 | 1.000 |
|  | Recovered | 20 | .441 | .429 | .453 |  |  |  |  | .00083 | .00081 | .00084 |  |  |  |
|  | Acute | 14 | .388 | .359 | .417 |  |  |  |  | .00094 | .00088 | .00101 |  |  |  |
| STR R | Controls | 12 | .388 | .377 | .399 | 1.000 | 1.000 | 1.000 |  | .00086 | .00084 | .00089 | 1.000 | 1.000 | 1.000 |
|  | Recovered | 20 | .387 | .381 | .393 |  |  |  |  | .00084 | .00083 | .00085 |  |  |  |
|  | Acute | 14 | .389 | .372 | .406 |  |  |  |  | .00087 | .00082 | .00093 |  |  |  |
| MdLF R | Controls | 12 | .404 | .394 | .415 | .011 | .003 | 1.000 |  | .00084 | .00082 | .00085 | .006 | <.001 | 1.000 |
|  | Recovered | 20 | .404 | .397 | .411 |  |  |  |  | .00082 | .00081 | .00083 |  |  |  |
|  | Acute | 14 | .380 | .369 | .392 |  |  |  |  | .00089 | .00086 | .00092 |  |  |  |
| AF R | Controls | 12 | .395 | .389 | .402 | .064 | .001 | 1.000 |  | .00080 | .00079 | .00081 | .102 | <.001 | 1.000 |
|  | Recovered | 20 | .401 | .393 | .409 |  |  |  |  | .00078 | .00077 | .00079 |  |  |  |
|  | Acute | 14 | .372 | .359 | .386 |  |  |  |  | .00084 | .00081 | .00086 |  |  |  |
| AR R | Controls | 12 | .408 | .387 | .428 | 1.000 | .736 | 1.000 |  | .00084 | .00080 | .00087 | 1.000 | .035 | 1.000 |
|  | Recovered | 20 | .417 | .398 | .436 |  |  |  |  | .00081 | .00079 | .00083 |  |  |  |
|  | Acute | 13 | .392 | .369 | .415 |  |  |  |  | .00088 | .00083 | .00092 |  |  |  |
| ATR R | Controls | 12 | .388 | .381 | .395 | 1.000 | .333 | 1.000 |  | .00081 | .00079 | .00082 | .016 | <.001 | 1.000 |
|  | Recovered | 20 | .393 | .386 | .400 |  |  |  |  | .00079 | .00078 | .00080 |  |  |  |
|  | Acute | 14 | .378 | .364 | .392 |  |  |  |  | .00090 | .00085 | .00095 |  |  |  |
| CBD R | Controls | 12 | .378 | .364 | .392 | 1.000 | 1.000 | 1.000 |  | .00079 | .00078 | .00081 | .141 | .002 | 1.000 |
|  | Recovered | 20 | .383 | .372 | .394 |  |  |  |  | .00078 | .00076 | .00079 |  |  |  |
|  | Acute | 14 | .362 | .336 | .387 |  |  |  |  | .00083 | .00080 | .00086 |  |  |  |

Supplementary Table 7: **Overview of the results of probabilistic tractography (fractional anisotropy and mean diffusivity) after adjustment of data in order to minimize scanner effects based on the validational cohort.** Mean values are shown for each tract for the group of controls, recovered and acute cases as well as 95% confidence intervals. P-values of post-hoc t-tests adjusted using Bonferroni correction for multiple testing are shown for comparisons between acute cases and controls, acute and recovered cases as well as recovered cases and controls. Abbreviations: B = bilateral, L = left, R = right. AC = Anterior Commissure; AF= Arcuate Fasciculus; AR= Acoustic Radiation; ATR = Anterior Thalamic Radiation; CBD = dorsal Cingulum subsection; CBP = peri-genual Cingulum subsection; CBT = temporal Cingulum subsection; CST = Corticospinal Tract; FAs = Frontal Aslant; FMA = Forceps Major; FMI = Forceps Minor; FX = Fornix; ILF = Inferior Longitudinal Fasciculus; IFO = Inferior Fronto-Occipital Fasciculus; MCP = Middle Cerebellar Peduncle; MdLF = Middle Longitudinal Fasciculus; OR = Optic Radiation; SLF1/ 2/ 3 = Superior Longitudinal Fasciculus 1/ 2/ 3; STR = Superior Thalamic Radiation; UF = Uncinate Fasciculus; VOF = Vertical Occipital Fasciculus.


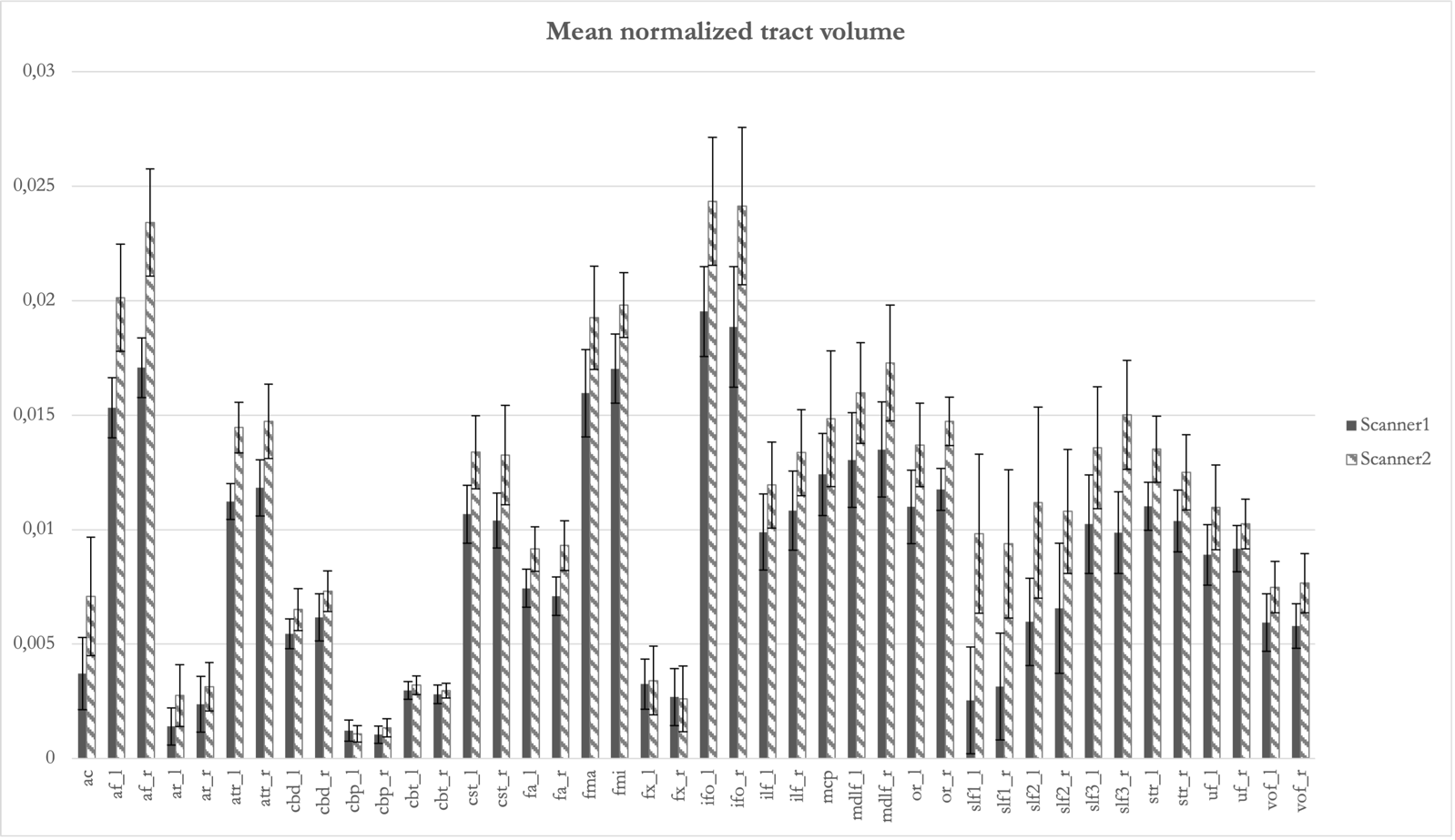


Supplementary Figure 1: **Overview over mean normalized tract volumes for the validational cohort used to correct for scanner artifacts.** Due to pandemic reasons, two scanners were used within this study: Scanner 1 (dark grey bars) represents the data from the 3T SIEMENS Skyra scanner, scanner 2 (striped bars) represents the data from the 1.5T SIEMENS Aera scanner. Mean values for the validational cohort are shown for each tract. Error bars indicate standard deviations. Abbreviations: B = bilateral, L = left, R = right. AC = Anterior Commissure; AF= Arcuate Fasciculus; AR= Acoustic Radiation; ATR = Anterior Thalamic Radiation; CBD = dorsal Cingulum subsection; CBP = peri-genual Cingulum subsection; CBT = temporal Cingulum subsection; CST = Corticospinal Tract; FAs = Frontal Aslant; FMA = Forceps Major; FMI = Forceps Minor; FX = Fornix; ILF = Inferior Longitudinal Fasciculus; IFO = Inferior Fronto-Occipital Fasciculus; MCP = Middle Cerebellar Peduncle; MdLF = Middle Longitudinal Fasciculus; OR = Optic Radiation; SLF1/ 2/ 3 = Superior Longitudinal Fasciculus 1/ 2/ 3; STR = Superior Thalamic Radiation; UF = Uncinate Fasciculus; VOF = Vertical Occipital Fasciculus.


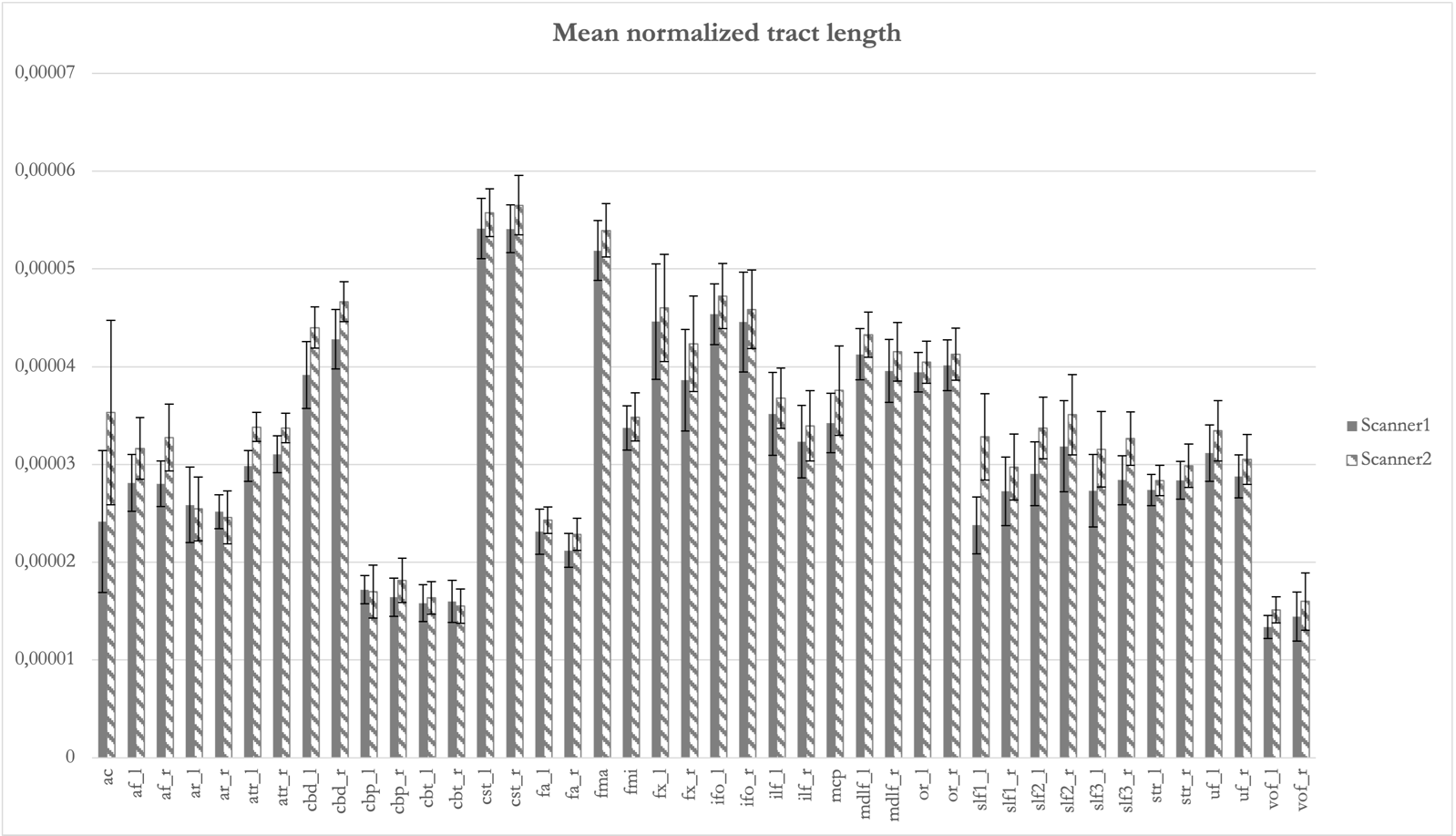


Supplementary Figure 2: **Overview over mean normalized tract lengths for the validational cohort used to correct for scanner artifacts.** Due to pandemic reasons, two scanners were used within this study: Scanner 1 (dark grey bars) represents the data from the 3T SIEMENS Skyra scanner, scanner 2 (striped bars) represents the data from the 1.5T SIEMENS Aera scanner. Mean values for the validational cohort are shown for each tract. Error bars indicate standard deviations. Abbreviations: B = bilateral, L = left, R = right. AC = Anterior Commissure; AF= Arcuate Fasciculus; AR= Acoustic Radiation; ATR = Anterior Thalamic Radiation; CBD = dorsal Cingulum subsection; CBP = peri-genual Cingulum subsection; CBT = temporal Cingulum subsection; CST = Corticospinal Tract; FAs = Frontal Aslant; FMA = Forceps Major; FMI = Forceps Minor; FX = Fornix; ILF = Inferior Longitudinal Fasciculus; IFO = Inferior Fronto-Occipital Fasciculus; MCP = Middle Cerebellar Peduncle; MdLF = Middle Longitudinal Fasciculus; OR = Optic Radiation; SLF1/ 2/ 3 = Superior Longitudinal Fasciculus 1/ 2/ 3; STR = Superior Thalamic Radiation; UF = Uncinate Fasciculus; VOF = Vertical Occipital Fasciculus.


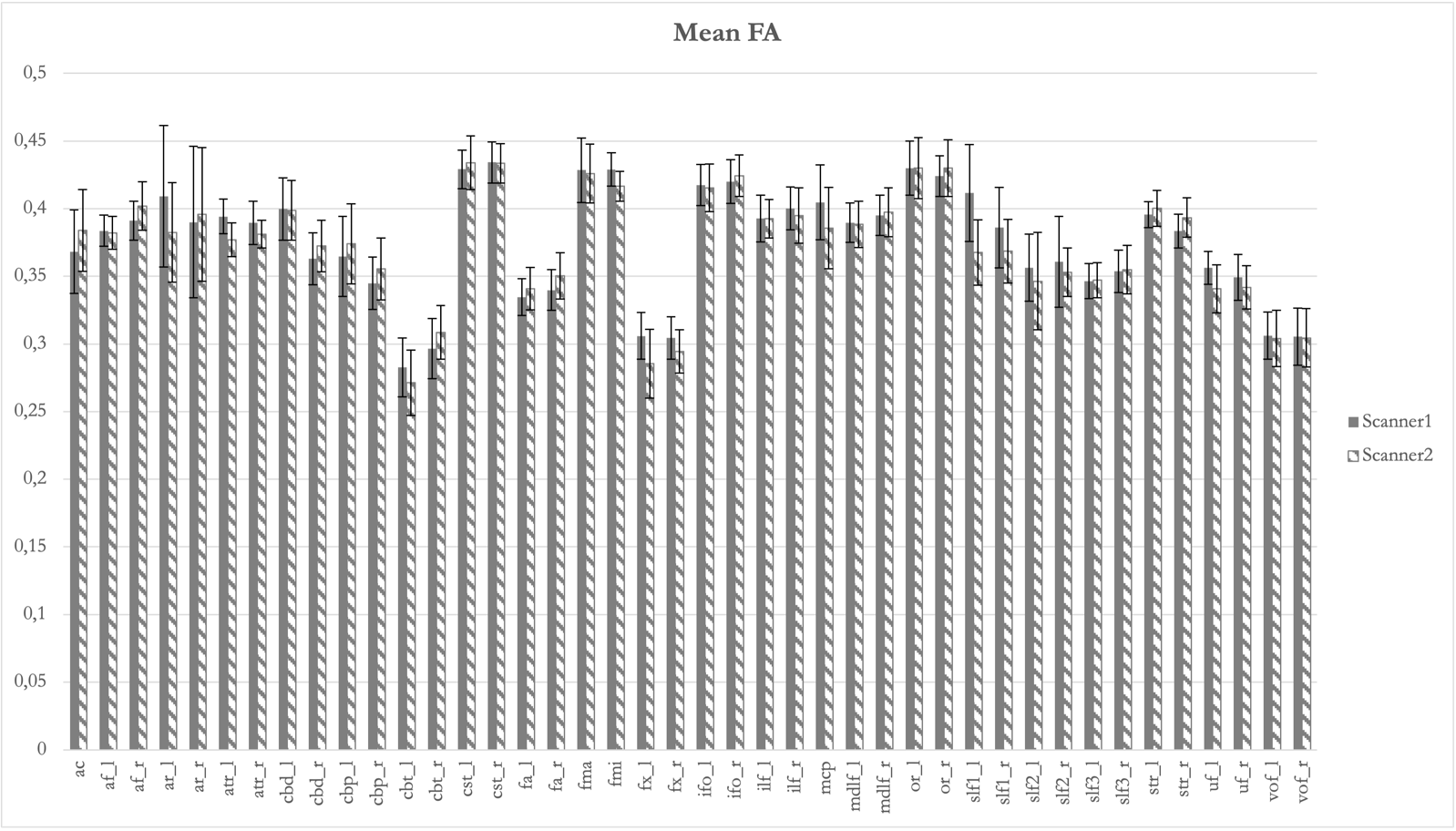


Supplementary Figure 3: **Overview over mean Fractional Anisotropy (FA) for the validational cohort used to correct for scanner artifacts.** Due to pandemic reasons, two scanners were used within this study: Scanner 1 (dark grey bars) represents the data from the 3T SIEMENS Skyra scanner, scanner 2 (striped bars) represents the data from the 1.5T SIEMENS Aera scanner. Mean values for the validational cohort are shown for each tract. Error bars indicate standard deviations. Abbreviations: B = bilateral, L = left, R = right. AC = Anterior Commissure; AF= Arcuate Fasciculus; AR= Acoustic Radiation; ATR = Anterior Thalamic Radiation; CBD = dorsal Cingulum subsection; CBP = peri-genual Cingulum subsection; CBT = temporal Cingulum subsection; CST = Corticospinal Tract; FAs = Frontal Aslant; FMA = Forceps Major; FMI = Forceps Minor; FX = Fornix; ILF = Inferior Longitudinal Fasciculus; IFO = Inferior Fronto-Occipital Fasciculus; MCP = Middle Cerebellar Peduncle; MdLF = Middle Longitudinal Fasciculus; OR = Optic Radiation; SLF1/ 2/ 3 = Superior Longitudinal Fasciculus 1/ 2/ 3; STR = Superior Thalamic Radiation; UF = Uncinate Fasciculus; VOF = Vertical Occipital Fasciculus.


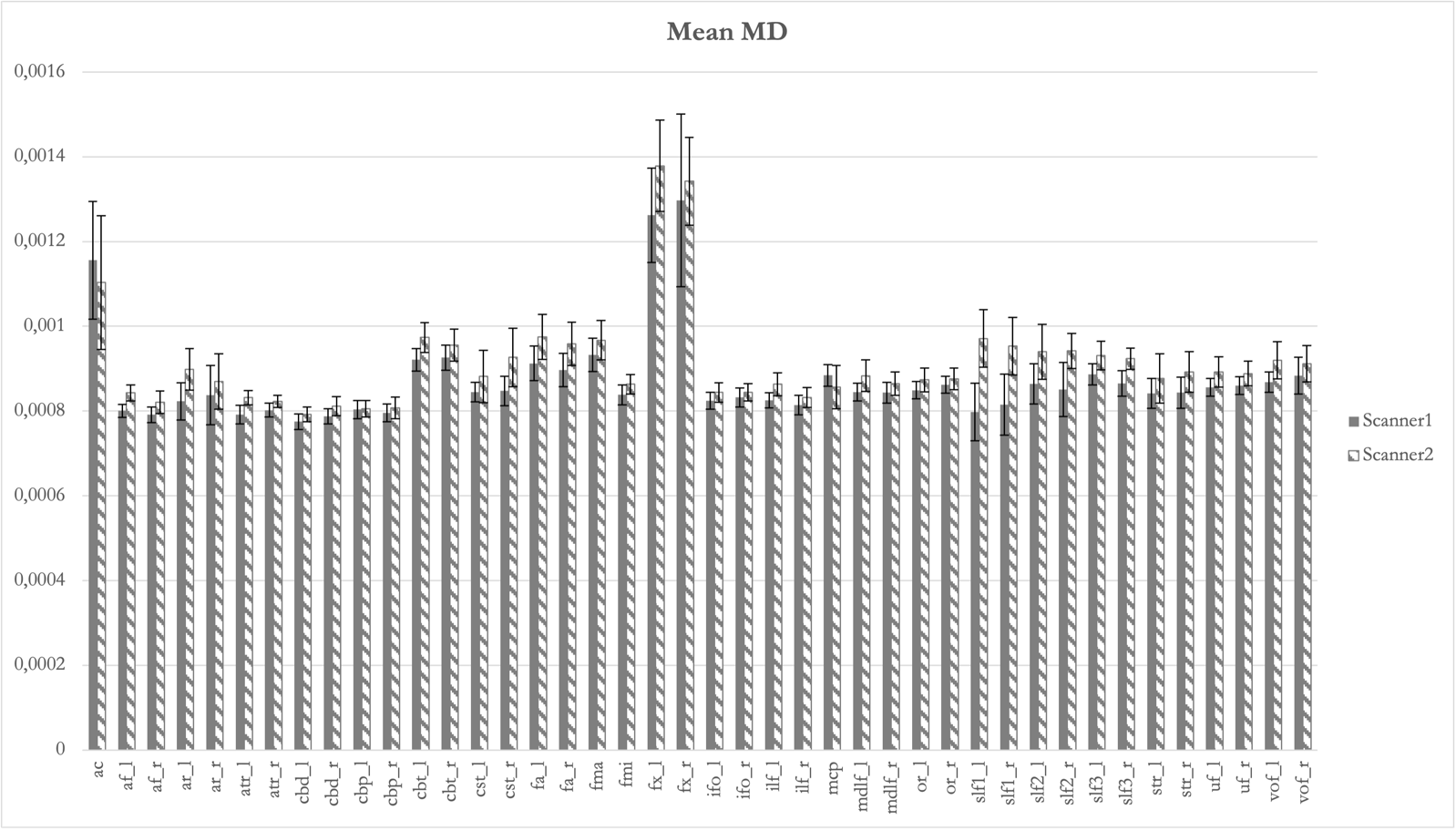


Supplementary Figure 4: **Overview over mean Mean Diffusivity (MD) for the validational cohort used to correct for scanner artifacts.** Due to pandemic reasons, two scanners were used within this study: Scanner 1 (dark grey bars) represents the data from the 3T SIEMENS Skyra scanner, scanner 2 (striped bars) represents the data from the 1.5T SIEMENS Aera scanner. Mean values for the validational cohort are shown for each tract. Error bars indicate standard deviations. Abbreviations: B = bilateral, L = left, R = right. AC = Anterior Commissure; AF= Arcuate Fasciculus; AR= Acoustic Radiation; ATR = Anterior Thalamic Radiation; CBD = dorsal Cingulum subsection; CBP = peri-genual Cingulum subsection; CBT = temporal Cingulum subsection; CST = Corticospinal Tract; FAs = Frontal Aslant; FMA = Forceps Major; FMI = Forceps Minor; FX = Fornix; ILF = Inferior Longitudinal Fasciculus; IFO = Inferior Fronto-Occipital Fasciculus; MCP = Middle Cerebellar Peduncle; MdLF = Middle Longitudinal Fasciculus; OR = Optic Radiation; SLF1/ 2/ 3 = Superior Longitudinal Fasciculus 1/ 2/ 3; STR = Superior Thalamic Radiation; UF = Uncinate Fasciculus; VOF = Vertical Occipital Fasciculus.


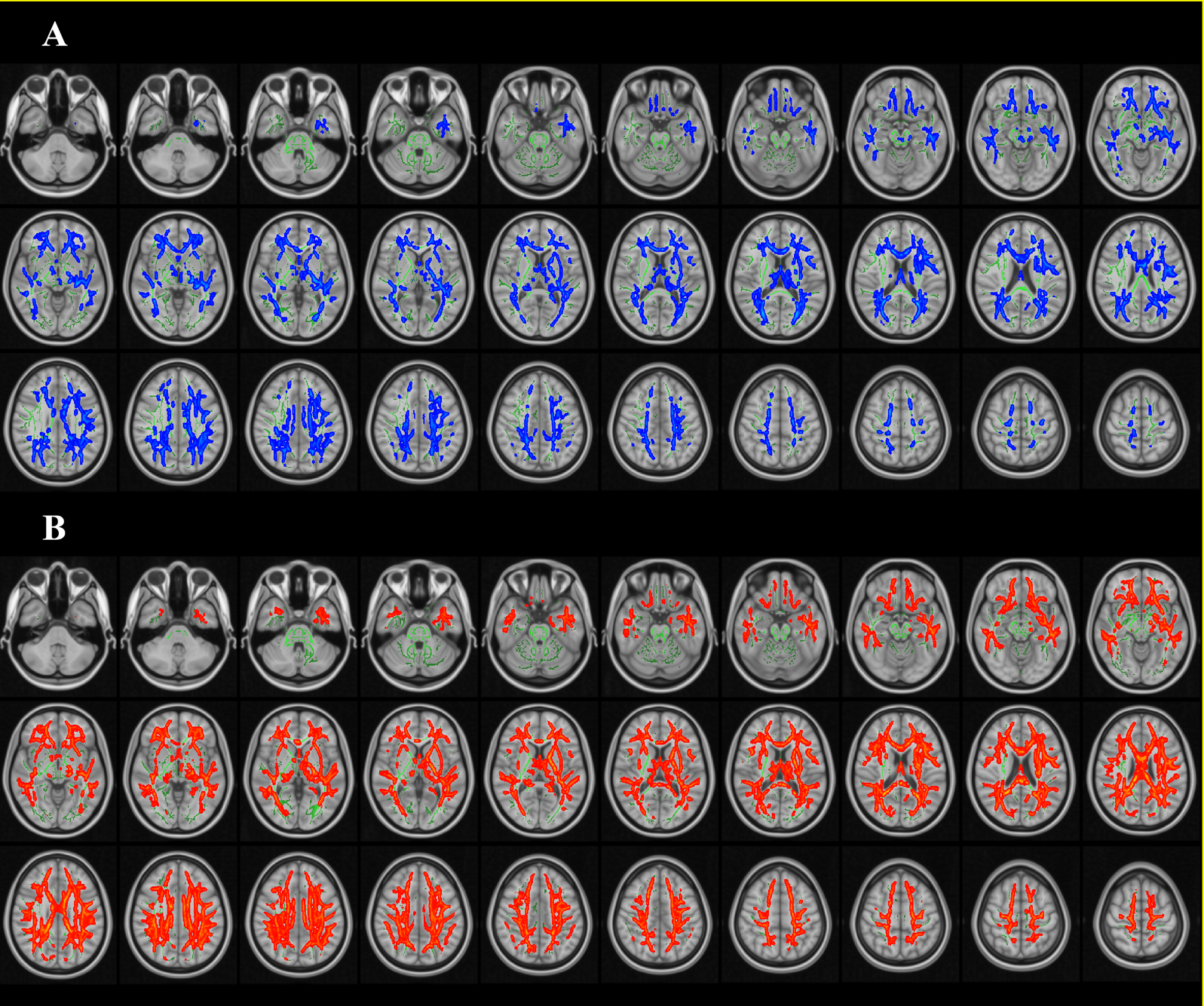


Supplementary Figure 5: **Overview of results from TBSS comparing acute patients with controls: A)** Fractional Anisotropy (FA), **B)** Mean Diffusivitiy (MD). Results are shown as TFCE (Threshold-Free Cluster Enhancement) p-value images after full correction for multiple comparisons across space for a p-value of 0.05. Blue colours represent a negative direction of differences (acute patients < controls), red colours indicate a positive direction of differences (acute patients > controls). The mean FA skeleton is shown in green.


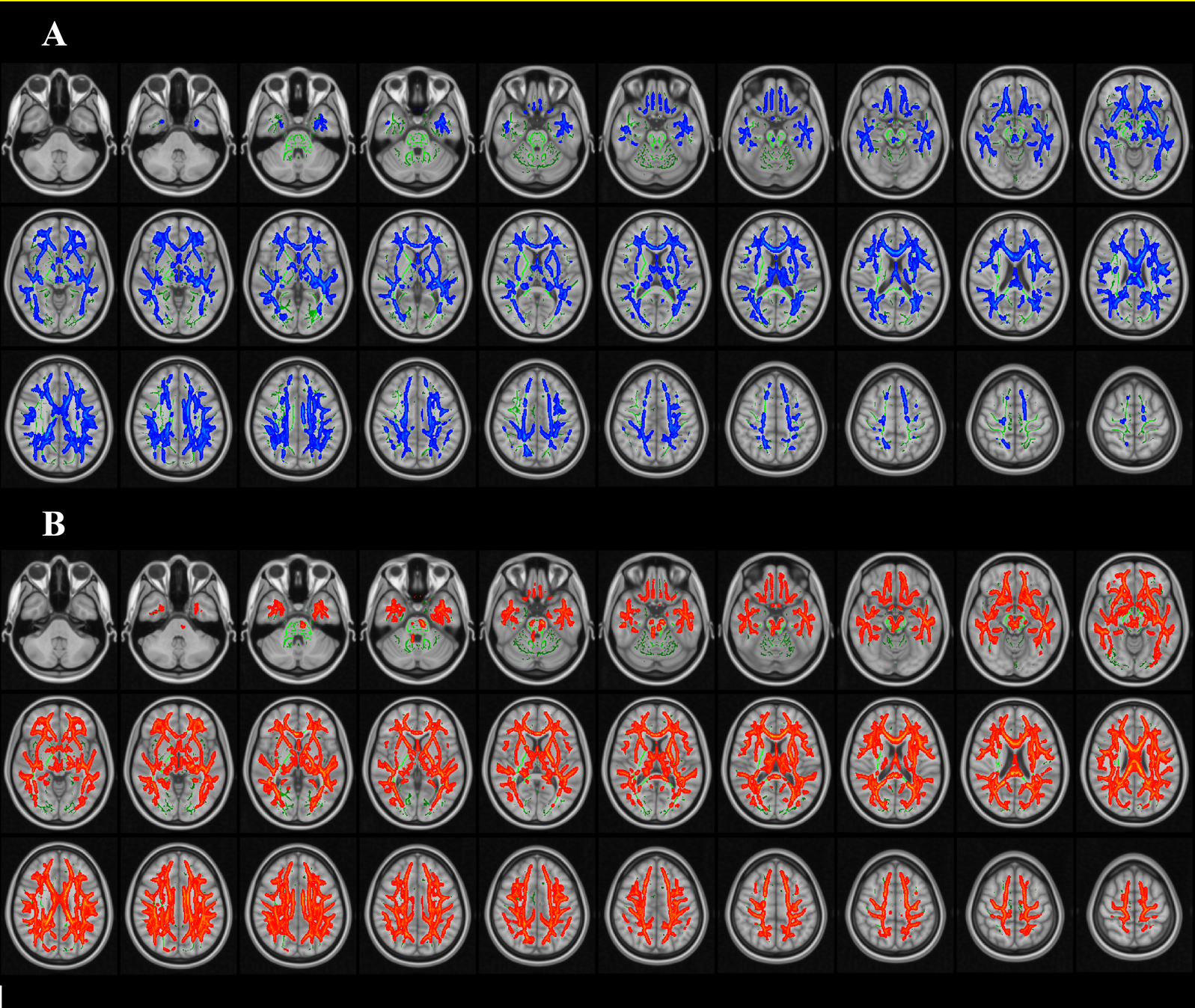


Supplementary Figure 6: **Overview of results from TBSS comparing acute patients with recovered: A)** Fractional Anisotropy (FA), **B)** Mean Diffusivitiy (MD). Results are shown as TFCE (Threshold-Free Cluster Enhancement) p-value images after full correction for multiple comparisons across space for a p-value of 0.05. Blue colours represent a negative direction of differences (acute patients < recovered), red colours indicate a positive direction of differences (acute patients > recovered). The mean FA skeleton is shown in green.


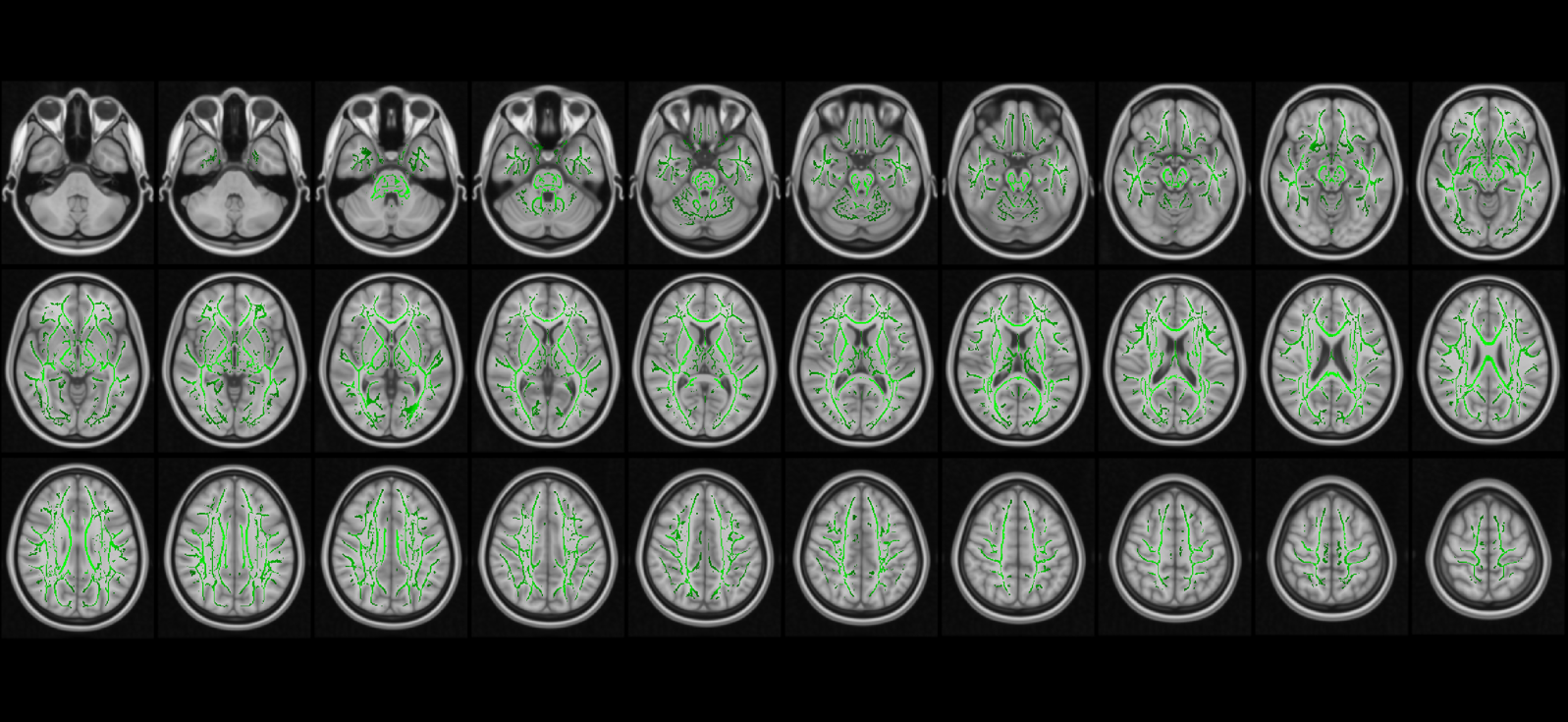


Supplementary Figure 7: **Overview of results from TBSS comparing recovered patients with controls:** Regarding both Fractional Anisotropy (FA) and Mean Diffusivitiy (MD), no significant differences were found between the groups. Therefore, TFCE (Threshold-Free Cluster Enhancement) p-value images show no significant areas after full correction for multiple comparisons across space for a p-value of 0.05. The mean FA skeleton is shown in green.
